# MtrAB activates ectoine production and triggers sporulation in response to osmotic stress in Streptomyces venezuelae

**DOI:** 10.1099/mic.0.001706

**Published:** 2026-05-20

**Authors:** Ainsley D.M. Beaton, Rebecca Devine, Neil A. Holmes, Nicolle F. Som, Lucas Balis, Katie Noble, Rhea Stringer, Martin Rejzek, Gerhard Saalbach, Barrie Wilkinson, Matthew I. Hutchings

**Affiliations:** 1 Department of Molecular Microbiology, John Innes Centre, Norwich Research Park, Norwich, NR4 7UH, UK; 2 Centre for Microbial Interactions, Norwich Research Park, NR4 7UG, Norwich, UK; 3 Cell and Developmental Biology Department, John Innes Centre, Norwich Research Park, Norwich NR4 7UH, UK; 4 Department of Biochemistry and Metabolism, John Innes Centre, Norwich Research Park, Norwich NR4 7UH, UK

**Keywords:** BldM, MtrA, osmotic stress, response regulators, sporulation, *Streptomyces*, WhiI

## Abstract

The MtrAB two-component system is a master regulator of antibiotic biosynthesis in *Streptomyces* species. MtrA is also required for sporulation under certain growth conditions, which means that on some growth media, *∆mtrA* mutant colonies do not produce aerial hyphae or spores. These mutants are referred to as (conditionally) bald because they lack the hairy appearance of wild-type colonies. Here, we report that *Streptomyces venezuelae* NRRL B-65442 *∆mtrA* is bald on R2YE agar but sporulates normally on MYM (Maltose Yeast Extract Medium) agar, and we demonstrate that this is caused by the presence of 10.2% sucrose in R2YE agar. Consistent with this, we found that adding 10.2% sucrose to MYM agar also inhibits sporulation of the *∆mtrA* mutant. Proteomics combined with DNA binding studies revealed that MtrA directly activates the expression of the key developmental regulator genes *bldM* and *whiI* on R2YE but not MYM agar. BldM and WhiI work together to activate genes required for aerial hyphae production and sporulation. Crucially, over-expression of *bldM-whiI* in the *∆mtrA* mutant restored normal sporulation in the presence of 10.2% sucrose. We hypothesized that MtrAB must sense and respond to osmotic stress, and consistent with this, we found that *∆mtrA* and *∆mtrB* mutants are bald on MYM agar containing 0.5 M NaCl. MtrA also directly activates biosynthesis of the compatible solute and osmoprotectant ectoine on growth media containing 10.2% sucrose. We propose a model in which high concentrations of sucrose or salt induce hyperosmotic stress in *Streptomyces* species, and this activates MtrAB. The response regulator MtrA then directly activates expression of the *ectABCD* operon to switch on ectoine biosynthesis and expression of *bldM* and *whiI* to trigger entry into sporulation.

**Impact Statement**

*Streptomyces* species have complex developmental life cycles that start with spore germination and outgrowth of an actively growing substrate mycelium. Environmental stresses, including nutrient starvation and osmotic stress, trigger the production of aerial hyphae that undergo cell division to form spores. These spores are more resistant to environmental stresses and can stay viable in the soil until conditions improve. Here, we show that MtrAB is responsible for sensing and responding to osmotic stress, and we demonstrate that it directly controls the biosynthesis of ectoine and the developmental transition to sporulation through direct activation of BldM and WhiI. This work provides new insight into how environmental cues control the life cycle of *Streptomyces* bacteria.

## Data Summary

The *Streptomyces venezuelae* NRRL B-65442 genome sequence is available at National Center for Biotechnology Information (NCBI) (Reference Sequence NZ_CP018074.1) and can be viewed at http://strepDB.streptomyces.org.uk (select vnz chromosome from the drop-down list). The ChIP-seq data (GEO accession number CP018074) used to map MtrA binding sites on the *S. venezuelae* NRRL B-65442 chromosome and the differential RNA-seq data (accession number GSE81104) used to map global transcript start sites throughout the life cycle were generated for earlier studies [1,2]. The tandem mass tag proteomics data are available via ProteomeXchange with identifier PXD069151. The ReDCaT SPR data are included in the manuscript and the supplementary information. Protocols are freely available at http://actinobase.org [3], and strains and plasmids are available from https://streptomyces.org.uk/ . The authors confirm all supporting data, code and protocols have been provided within the article or through supplementary data files.

## Introduction

The genus *Streptomyces* comprises >1,100 verified species whose specialised metabolites form the basis of around 55% of clinically used antibiotics [4,5]. They are ubiquitous in soils where they play an important role in the turnover of organic material and are enriched in the rhizosphere and endosphere of many different plants [6,9]. Their complex developmental life cycles include hyphal growth and sporulation, and these are often linked to the production of bioactive specialised metabolites [4,10]. These structurally diverse specialised metabolites play important roles in their interactions with other soil organisms, including microbes, insects and plants [11,12]. Given the diversity of environments they colonise, it is perhaps not surprising that more than 20% of the genes in their large (6–12 Mbp) linear genomes encode transcription factors and signal transduction systems, while up to 10% of their genomes are dedicated to specialised metabolism [13].

This study focuses on the model organism *Streptomyces venezuelae* NRRL B-65442 [5], which encodes 57 two-component signal transduction systems (TCS), with 15 of these conserved throughout the genus [14]. TCS allow bacteria to sense and respond to extracellular signals, and the MtrAB system is highly conserved both within *Streptomyces* species and across the wider phylum Actinomycetota (formerly Actinobacteria) [15]. The *mtrAB* operon also includes the *lpqB* gene, encoding a lipoprotein which has been shown to modulate MtrB sensor kinase activity in *Mycobacterium smegmatis* [15,16]. The response regulator MtrA was first identified in mycobacteria (Mycobacterial transcriptional regulator A) and is essential in *Mycobacterium tuberculosis* [17], where it coordinates DNA replication with cell division [18,20]. In *Corynebacterium glutamicum*, MtrAB senses osmotic stress and controls a regulon of genes encoding products involved in the osmotic stress response, including the proline transporter ProP [21]. In *Streptomyces* species, MtrA has been implicated in the regulation of antibiotic production and development, including sporulation [22], and binds to at least some of the same sites as the atypical response regulator GlnR to coordinate the regulation of nitrogen metabolism [23,24]. MtrA has also been implicated in the regulation of antibiotic production in rare actinomycetes, including erythromycin in *Saccharopolyspora erythraea* [25,26]. MtrA directly regulates the production of antibiotics in *Streptomyces coelicolor* and *S. venezuelae* and coordinates the production of chloramphenicol with sporulation in *S. venezuelae* NRRL B-65442, such that loss of MtrA activity leads to constitutive high-level production of this antibiotic [1,27, 28]. Deletion of *mtrA* also leads to a conditional bald phenotype in these species, where they cannot produce aerial hyphae or sporulate under certain growth conditions. MtrA also represses the production of avermectin in *Streptomyces avermitilis* while activating morphological differentiation [29]. Thus, modulation of MtrAB activity can be used to increase the production of antibiotics in *Streptomyces* species and other closely related filamentous actinomycetes. Consistent with this, MtrA binding sites have been identified within biosynthetic gene clusters for medically important compounds, including gentamicin, rifamycin, oxytetracycline, daptomycin and streptomycin [28]. It appears that MtrA directly controls antibiotic production in these bacteria, but we do not understand how MtrA controls development or why *∆mtrA* mutants are conditionally bald. This means they form aerial hyphae and spores on some growth media, but not others, and the reasons for this are not known.

In this work, we set out to address this question by comparing MtrA-dependent developmental gene expression in the wild-type and *∆mtrA* mutant strains grown on R2YE agar (without glucose), where *∆mtrA* is bald, and MYM (Maltose Yeast Extract Medium) agar where both strains sporulate normally. R2YE agar contains 10.2% sucrose, and we demonstrate that omitting sucrose from R2YE enables the *∆mtrA* mutant to sporulate, while adding 10.2% sucrose to MYM agar prevents sporulation. Thus, high sucrose concentrations appear to activate MtrAB activity, most likely by inducing osmotic stress, and this triggers sporulation such that an *∆mtrA* mutant cannot sporulate under these conditions. Next, we used surface plasmon resonance (SPR) to identify the exact MtrA binding sites at the promoters of the developmental genes *adpA*, *bldM*, *dnaA*, *filP*, *ssgB* and *whiI,* which were previously identified as MtrA targets using ChIP-seq [1] (Fig. S1, available in the online Supplementary Material). Quantitative tandem mass tag (TMT) proteomics showed that levels of the atypical response regulators BldM and WhiI are reduced fourfold and sevenfold, respectively, in the *∆mtrA* mutant grown on R2YE agar but not on MYM agar, while the other gene products were unaffected. Homodimeric BldM controls the expression of genes required for the development of aerial hyphae, while heterodimeric BldM-WhiI controls the expression of genes required for cell division of the aerial hyphae into spores [30]. Over-expression of *bldM–whiI* restored sporulation to the *∆mtrA* mutant under osmotic stress conditions. We thus hypothesise that MtrAB senses and responds to osmotic stress by triggering entry into sporulation. Consistent with this, we show that *∆mtrA* and *∆mtrB* mutants are bald on MYM agar containing 0.5 M NaCl and MtrA directly activates expression of the *ectABCD* operon, which encodes the biosynthetic pathway for the osmoprotectant ectoine, under hyperosmotic stress conditions [31]. Ectoine is produced at elevated levels on MYM and R2YE agar containing 10.2% sucrose, and this is dependent on MtrA. Our data support a model in which the osmotic stress response is mediated by MtrAB and results in the production of ectoine and spores, thus ensuring the survival of these bacteria until conditions improve.

## Methods

For detailed written and video protocols, visit http://actinobase.org [3].

### Strains, plasmids and primers

All the bacterial strains used in this work are listed in Table 1, and plasmids are listed in Table 2. The *∆mtrB* mutant was generated for a previous study [1]. Plasmids that were generated for this work were constructed as follows: DNA fragments containing 25–35 nt overlapping regions were assembled into digested DNA vectors using the exonuclease-based Gibson Assembly (NEB). A standard 3 : 1 ratio of insert to vector was used, and assembly was performed at 50 °C for 1 h in a thermocycler. All the primers used for cloning in this work are listed in Table S1.

**Table 1.** Bacterial strains used in this work

Bacterial strain	Description	Supplier and/or reference
*E. coli* DH5alpha	*E. coli* strain used for cloning	Thermo Fisher
*E. coli* Top10	F– *mcr*A Δ(*mrr-hsdRMS-mcrBC*) Φ80*lac*ZΔM15 Δ*lac*X74 *rec*A1 *ara*D139 Δ(*ara* leu) 7697 *gal*U *gal*K *rps*L (StrR) *end*A1 *nup*G	Invitrogen
*E. coli* ET12567/pUZ8002	A methylation-deficient (*∆dcm∆dam*) strain of *E. coli* containing the driver plasmid pUZ8002 for conjugation to *Streptomyces venezuelae*.	John Innes Centre (http://streptomyces.org.uk)
*E. coli* BL21+pETDuet-*mtrA*	*E. coli* expression strain used for purification of 6× His-MtrA *fhuA2 [lon] ompT gal [dcm] ΔhsdS*	New England Biolabs
*S. venezuelae* NRRL B-65442	Wild-type *S. venezuelae* NRRL B-65442	John Innes Centre (http://streptomyces.org.uk)
*S. venezuelae ∆mtrA*	*S. venezuelae* NRRL B-65442 with an in-frame deletion of *mtrA* generated using pCRISPomyces-2	This work
*S. venezuelae ∆mtrA +mtrA*	*S. venezuelae ∆mtrA* complemented in trans by integration of pSS170 containing *mtrA* under the control of the native promoter	This work
*S. venezuelae ∆mtrA* +PermE*-*bldM-whiI*	*S. venezuelae ∆mtrA* containing integrated pIJ10257 with an artificial *bldM-whiI* operon under the control of the high-level constitutive ermE* promoter.	This work
*S. venezuelae ∆mtrB*	*S. venezuelae* NRRL B65442 *∆mtrB* (with an in-frame deletion of *mtrA* generated using pCRISPomyces-2)	This work
*S. venezuelae ∆mtrB +mtrB*	*S. venezuelae* NRRL B65442 ∆*mtrB* + *mtrAp-mtrB*	This work

**Table 2.** Plasmids used in this work

Plasmid	Description	Resistance	Reference
pCRISPomyces-2	*AprR, oriT, reppSG5(ts), oriColE1, sSpcas9,* synthetic guide RNA cassette	Apramycin	RRID:Addgene_61737 [32]
pCRISP-*mtrA*	pCRISPomyces-2 containing the guide RNA to target *mtrA* plus repair templates that flank either end of the *mtrA* gene	Apramycin	This work
pSS170	Derivative of pMS82 [52], which lacks the apramycin promoter fragment and contains a multiple cloning site	Hygromycin	John Innes Centre (http://streptomyces.org.uk) [53]
pIJ10257	pMS81 [52] containing a 330 bp ermE* promoter fragment with RBS and multicloning site from pIJ8723 cloned in using KpnI-NsiI. Integrates into a single copy into the phiBT1 phage integration site in *Streptomyces* genomes	Hygromycin	John Innes Centre (http://streptomyces.org.uk) [54]
pSS170+*mtrA*	pSS170 with *mtrA* under the control of the *native* promoter. *mtrA* was amplified with primers NS300 and NS301 and ligated into pSS170 using HindIII	Hygromycin	This work
pIJ10257+*bldM-whiI*	pIJ10257 with *bldM-whiI* under the control of the ermE* promoter. *bldM* and *whiI* were amplified using primers RD001-RD004, which generated a continuous operon with an additional ribosome binding site between the two to drive expression of both genes under PermE*.	Hygromycin	This work
pETDuet	pETDuet has two multiple cloning sites (MCS): an N-terminal His•Tag in MCS1, f1 origin, T7 *lac*, *lacI, ori*	Ampicillin	Novagene
pETDuet+*mtrA*	Expression vector for over-producing 6xHis-tagged MtrA in *E. coli*. *mtrA* was amplified with primers NS145 and NS146 and ligated into pETDuet using BamHI and HindIII	Ampicillin	This work
pETDuet+*mtrA* D53A	Expression vector for over-producing 6xHis-tagged MtrA D53A in *E. coli*. Mutated *mtrA* was amplified with primers NS365 and NS366 and ligated into pETDuet using BamHI and HindIII	Ampicillin	This work
pETDuet+*mtrA* D53E	Expression vector for over-producing 6xHis-tagged MtrA D53E in *E. coli*. Mutated *mtrA* was amplified with primers NS365 and NS366 and ligated into pETDuet using BamHI and HindIII	Ampicillin	This work

### Growth media and conditions

The growth media used in this work are listed in Table 3. Recipes are also available at https://actinobase.org/index.php?title=Media_recipes. The final concentrations of antibiotics used for selection of plasmid carriage were 50 µg ml^−1^ of apramycin or 50 µg ml^−1^ hygromycin. Spores were pregerminated at 50 °C for 10 min in 2× yeast tryptone prior to conjugation with *Escherichia coli*. After conjugation, 25 µg ml^−1^ of nalidixic acid was used to kill the *E. coli* cells alongside the relevant antibiotic to select for *S. venezuelae* exconjugants.

**Table 3.** Growth media and supplements used for bacterial culturing

Media	Recipe (per litre)	Notes	Water	pH
MYM agar and broth	4 g maltose, 4 g yeast extract, 10 g malt extract, 2 ml trace element solution, ±20 g agar	For routine growth of *S. venezuelae* NRRL B-65442	50 : 50 tap/deionised	7.2
MYM agar+sucrose	As above, with an additional 103 g of sucrose		50 : 50 tap/deionised	7.2
R2YE agar without glucose	Dissolve 103 g sucrose, 0.25 g K_2_SO_4_, 10.12 g MgCl_2_.6H_2_O, 0.1 g Difco Casamino acids in 800 ml distilled H_2_O. Aliquot 80 ml into 250-ml flasks and add 2.2 g agar before autoclaving. Then, add to each flask the following autoclaved solutions in the order listed: 5 ml Difco yeast extract (10% solution), 1 ml KH_2_PO_4_ (0.5% solution), 8 ml CaCl_2_.2H_2_O (3.68% solution), 1.5 ml l-proline (20% solution), 10 ml TES buffer (5.73%, adjusted to pH 7.2), 0.2 ml trace element solution, 0.5 ml 1 N NaOH (unsterilized)		Deionised	7.2
R2YE agar without sucrose	As above, with sucrose omitted		Deionised	7.2
Luria–Bertani agar and broth	10 g tryptone, 5 g yeast extract, 10 g NaCl ±20 g agar	NaCl excluded for hygromycin selection	Deionised	n/a
2× yeast tryptone (YT)	31 g 2× YT Broth powder (Formedium)	For spore germination prior to conjugations	Deionised	7.4
Trace elements solution (per litre)	40 mg ZnCl_2_ 200 mg FeCl_3_ . 6H_2_O 10 mg CuCl_2_ . 2H_2_O 10 mg MnCl_2_ . 4H_2_O 10 mg Na_2_B_4_O_7_ . 10H_2_O 10 mg (NH_4_)_6_Mo_7_O_24_ . 4H_2_O	Media supplement for MYM and R2YE media	Deionised	

### Deletion of *mtrA* using Cas9-mediated genome editing

To make an in-frame deletion of the *mtrA* gene a guide RNA was designed and then generated by annealing primers NS308 and NS309 and cloned into the pCRISPomyces-2 plasmid using Golden Gate [32]. The vector containing the guide RNA was then linearized using XbaI to clone in the repair templates (generated using primers NS304-307) using Gibson Assembly. The resulting vector was named pCRISP-*mtrA*. A detailed protocol for the use of pCRISPomyces-2 is available at https://actinobase.org/index.php?title=CRISPR/Cas9-mediated_genome_editing.

### Purification of MtrA

6xHis-tagged MtrA was over-produced in *E. coli* BL21 using pETDuet. Cultures were grown in 2 l Luria–Bertani, shaking at 37 °C, 200 r.p.m. for 4 h, then induced with 100 µM IPTG and incubated at 18 °C overnight. Cells were harvested by centrifugation and lysed by sonication on ice in 20 mM Tris pH 8, 75 nM NaCl, 0.1% v/v Triton-X, 10 µg ml^−1^ lysozyme and EDTA-free protease inhibitors. The soluble fraction was harvested by centrifugation at 18,000 r.p.m. for 20 min; this was then applied directly to a nickel affinity chromatography (HiTrap) column on an AKTApure in a refrigerated cabinet. The column had been equilibrated with wash buffer (80 mM Tris, pH 8, 200 mM NaCl, 1 mM TCEP, 20 mM imidazole). After further rounds of column washing to remove non-specifically bound proteins, MtrA was eluted from the column using wash buffer +350 mM imidazole, and fractions were analysed by SDS-PAGE to confirm the presence of the protein.

### Reusable DNA capture technology with surface plasmon resonance

Oligonucleotides (Table S2) were designed against key developmental promoters and annealed in HBS-EP+Buffer to make double-stranded probes. DNA probes were annealed to a streptavidin chip prepared with reusable DNA capture technology (ReDCaT) linker DNA using the Biocore 8K+SPR system in HBS-EP+Buffer and water. Oligos were flowed over the chip at 10 µl min^−1^ for a 60 s capture, followed by freshly purified MtrA protein to analyse DNA binding activity. Binding was recorded at both early and late time points, after 60 s contact, followed by 60 s dissociation. The chip was regenerated using a 60 s wash with 1 M NaCl/50 mM NaOH reagent to remove protein and DNA probes for the next cycle. Binding responses were normalized for the molecular weight of both DNA and protein.

### TMT proteomics

*S. venezuelae* colonies were grown on cellophane-covered MYM or R2YE – glucose agar plates as triplicate spots from 5 µl spores, all in duplicate. After 5 days at 30 °C, the mycelium was scraped into a 15-ml Falcon tube and resuspended in 10 ml cell lysis buffer [50 mM TEAB buffer pH 8.0, 150 mM NaCl, 2% SDS, EDTA-free protease inhibitor, PhosSTOP phosphatase inhibitor (Sigma-Aldrich)]. The suspension was disrupted via French press three times before being boiled for 10 min. Samples were sonicated at 50 kHz four times for 20 s per cycle and then pelleted at 3,220 ***g*** for 30 min. Protein concentration was determined using the BCA assay, and 1 mg of protein from each sample was transferred to a fresh 15-ml Falcon tube. Four volumes of methanol were added, and the sample was vortexed, and then 1 vol of chloroform and 3 volumes of distilled H_2_O were added. Samples were centrifuged at 4,000 ***g*** for 10 min, and the upper layer was discarded, leaving the interphase behind. Another four volumes of methanol was added to the bottom phase and then vortexed, followed by thorough vortexing and centrifugation at 4,000 ***g*** for 20 min. As much solvent as possible was removed without disturbing the pellet, and 3 ml of acetone was added. This was incubated for 10 min on the bench and then transferred to a microcentrifuge tube for centrifugation at 13,000 r.p.m. for 20 min. The acetone was removed, and the pellet was air-dried before continuing.

The protein pellets were resuspended in 250 µl of 2.5% sodium deoxycholate (Merck) in 0.2 M EPPS buffer (Merck), pH 8.5, and vortexed under heating. Protein concentration was estimated using a BCA assay. For the analysis, ~100 µg of protein per sample was used. The proteins were treated with DTT (10 mM, 60 °C, 45 min) and iodoacetamide (30 mM, 45 min) to reduce and alkylate cysteine residues. One µg of trypsin (Sequencing Grade, Promega) was added per sample, and the samples were incubated at 40 °C for about 18 h. After the digest, TMT labelling was performed using 12 channels from a TMT^™^ 16plex kit (Lot ZD386952, Thermo Fisher Scientific) according to the manufacturer’s instructions. Samples were assigned to the TMT channels in alternating order to avoid channel leakage according to Brenes *et al*. between different conditions, with channels 126, 130 N, 130 C, 131 N omitted [33]. After 2 h incubation, aliquots of 2 µl from each sample were combined in 600 µl 0.5% trifluoroacetic acid (TFA), desalted and analysed on the mass spectrometer (same method as for TMT, but without RTS; see below) to test labelling efficiency and estimate total sample abundances. The main sample aliquots were quenched by adding 8 µl of 5% hydroxylamine and then combined to roughly level abundances according to the test and desalted using a C18 Sep-Pak cartridge (200 mg, Waters). The eluted peptides were dissolved in 500 µl of 25 mM NH_4_HCO_3_ and fractionated by high pH-reversed phase HPLC. Using an ACQUITY Arc Bio System (Waters), the samples were loaded onto an XBridge^®^ 5 µm BEH C18 130 Å column (250×4.6 mm, Waters). Fractionation was performed with the following gradient of solvents A (water), B (acetonitrile) and C (25 mM NH_4_HCO_3_ in water, pH 8.5) at a flow rate of 1 ml min^−1^: solvent C was kept at 10% throughout the gradient; solvent B: 0–5 min: 5%, 5–10 min: 5–10%, 10–80 min: 10–45%, 80–90 min: 45–80%, followed by 5 min at 80% B and re-equilibration to 5% for 25 min. Fractions of 1 ml were collected and concatenated by combining distant fractions of similar peptide concentration to produce 23 final fractions for MS analysis. Aliquots were analysed by nanoLC-MS/MS on an Orbitrap Eclipse™ Tribrid™ mass spectrometer equipped with a FAIMS Pro Duo interface and coupled to a Vanquish™ Neo UHPLC System (Thermo Fisher Scientific, Hemel Hempstead, UK). The samples were loaded onto a trap cartridge (PepMap™ Neo Trap Cartridge, C18, 5 um, 0.3×5 mm, Thermo) with 10 µl of 0.1% TFA at 15 µl min^−1^. The trap column was then switched in-line with the analytical column (Aurora Frontier TS, 60 cm nanoflow UHPLC column, ID 75 µm, reversed phase C18, 1.7 µm, 120 Å; IonOpticks, Fitzroy, Australia) for separation at 60 °C using the following gradient of solvents A (water, 0.1% formic acid) and B (80% acetonitrile, 0.1% formic acid) at a flow rate of 0.25 µl min^−1^ : 0–7 min increase B from 1 to 7% (curve 4), 7–107 min increase B to 46% (or 52% depending on fraction); 107–112 min linear increase B to 99%; keeping at 99% B for 3 min and re-equilibration to 1% B. Data were acquired with the following parameters in positive ion mode: MS1/OT: resolution 120 k, profile mode, mass range *m/z* 400–1600, AGC target 4e^5^, max inject time 50 ms, FAIMS device set to three compensation voltages (−35V, −50V, −65V) for 1 s each; MS2/IT: for each CV, data dependent analysis with the following parameters: 1 s cycle time rapid mode, centroid mode, quadrupole isolation window 0.7 Da, charge states 2–5, threshold 1.9e^4^, CID CE=30, AGC target 1e4, max. inject time 50 ms, dynamic exclusion 1 count for 30 s, mass tolerance ±7 p.p.m.; MS3 synchronous precursor selection (SPS): 10 SPS precursors, isolation window 0.7 Da, HCD fragmentation with CE=50, Orbitrap Turbo TMT and TMTpro resolution 30 k, AGC target 200%, max inject time 100 ms; real-time search: protein database *S. venezuelae*, enzyme trypsin, 1 missed cleavage, oxidation (M) as variable, carbamidomethyl (C) and TMTpro as fixed modifications, precursor tolerance 10 p.p.m., match parameters: Xcorr=1.4, dCn=0.1.

The mass spectrometry raw data were processed and quantified in Proteome Discoverer 3.2 (Thermo); all mentioned tools of the following workflow are nodes of the proprietary Proteome Discoverer (PD) software. The *S. venezuelae database* was imported into PD, adding a reversed sequence database for decoy searches; a database for common contaminants (maxquant.org, 245 entries) was also included. The database search was performed using the incorporated search engines CHIMERYS (MSAID, Munich, Germany) and Comet [34]. The processing workflow included recalibration of MS1 spectra, reporter ion quantification by the most confident centroid (20 p.p.m.) and a search on the imported *S. venezuelae* database. The TopN Peak Filter was applied with 20 peaks per 100 Da. For CHIMERYS, the inferys_4.7.0._fragmentation prediction model was used with fragment tolerance of 0.3 Da, enzyme trypsin with one missed cleavage, variable modification oxidation (M), fixed modifications carbamidomethyl (C) and TMTpro on N-terminus and K. For Comet, the version 2019.01 rev.0 parameter file was used with default settings except precursor tolerance set to 5 p.p.m. and trypsin missed cleavages set to 1. Modifications were the same as for CHIMERYS. The consensus workflow included the following parameters: use of intensity-based abundance, normalization on total peptide abundances, protein abundance-based ratio calculation, unique peptides (protein groups) for quantification, TMT channel correction values applied (Lot ZD386952), co-isolation/SPS matches/CHIMERYS coefficient thresholds 50%/65%, 0.8; missing values imputation by low abundance resampling, hypothesis testing by t-test (background based), adjusted *P*-value calculation by the Benjamini–Hochberg procedure. The results were filtered for strict FDR confidence (0.01) and Master proteins and were exported into a Microsoft Excel table, including, among others, results for normalized and un-normalized abundances, ratios for the specified conditions calculated from the normalized abundances, the corresponding *P*-values and adjusted *P*-values, number of unique peptides, *q*-values, PEP-values and identification scores from both search engines.

### Ectoine extraction and quantification

Strains were grown in lawns for 3 days (MYM) or 7 days (R2YE), and small plugs of equal size were taken for extraction (*n*=3). Plugs were freeze-thawed to assist with cell lysis before being submerged in ice-cold 50 : 50 water/methanol (400 µl). Samples were sonicated on ice until lysed (15 s on, 15 s off, for 3 min) and incubated on ice for a further 20 min with occasional vortexing. Samples were then centrifuged at 13,000 r.p.m. for 10 min at 4 °C to pellet the cell debris. Supernatant was collected and dried in a GeneVac evaporator. Samples were resuspended in 90% acetonitrile with 10% dH_2_O+0.1% formic acid and diluted 1 : 10 for analysis. The samples were analysed by liquid chromatography coupled with electrospray ionization tandem mass spectrometry (LC-ESI-MS/MS) on a TQ-Absolute triple quadrupole mass spectrometer (Waters) operated in MRM (Multiple Reaction Monitoring) mode. ESI-MS/MS analysis was performed in positive ion mode using a source with a capillary voltage of 3 kV, 600 °C desolvation temperature, 900 l/h desolvation gas, 150 l/h cone gas and 7 bar nebulizer pressure. MRM transitions for ectoine standard (Sigma-Aldrich) in positive ESI mode (Table 4) were generated using IntelliStart software. A sample of ectoine (10 µM) was introduced at 10 µl min^−1^ combined with a flow from the UPLC pump typical of an LC run. Liquid chromatography was achieved on an Accucore 150-Amide-HILIC (100×2.1 mm, 2.6 µm) column equipped with a column guard. Ectoine was eluted using mobile phase A (water containing 0.1% formic acid) and mobile phase B (acetonitrile using the following multistep gradient at a flow rate 400 µl min^−1^): 0 min, 95% B; 1 min, 95% B; 10 min, 60% B; 11 min, 60% B; 12 min, 95% B; 20 min, 95% B. Ectoine standard (10 µM) was injected (1 µl) to determine retention time. Once the LC retention time of ectoine has been established, the mass transitions were collected in time windows centred on the relevant peak. MassLynx software (Waters) was used to collect, analyse and process data. The limit of detection for ectoine was determined to be 10 fmol on the column using a serial dilution. Transition 143 → 97 was used for quantification.

**Table 4.** MRM transitions of ectoine

Standard	MRM transition	Cone (V)	Collision energy (eV)	Dwell (s)
Ectoine	143 → 68 143 → 97	2.00 2.00	22.00 16.00	1.497 1.497

### Cryo-scanning electron microscopy

Samples were prepared for cryo-scanning electron microscopy (cryo-SEM) by streaking for single colonies on the appropriate media as described above and incubated at 30 °C for the indicated number of days. Samples were mounted on an aluminium stub using Tissue-Tek^R^ (BDH Laboratory Supplies, Poole, England). The stub was immediately plunged into liquid nitrogen slush (~−210 °C) to cryo-preserve the material. The frozen sample was then transferred to the cryo-stage of an ALTO 2500 cryo-transfer system (Gatan, Oxford, England) attached to an FEI Nova NanoSEM 450 (FEI, Eindhoven, the Netherlands).

Surface frost was sublimated at −95 °C for 4 min, after which the sample was sputter-coated with platinum for 130 s at 10 mA, at a temperature below −110 °C. The sample was then transferred to the main chamber of the microscope, where the cryo-stage was maintained at ~−125 °C. Imaging was performed at 3 kV, and digital TIFF files were recorded.

## Results and discussion

### Deletion of *S. venezuelae mtrA* prevents sporulation on R2YE agar

**Fig. 1.**
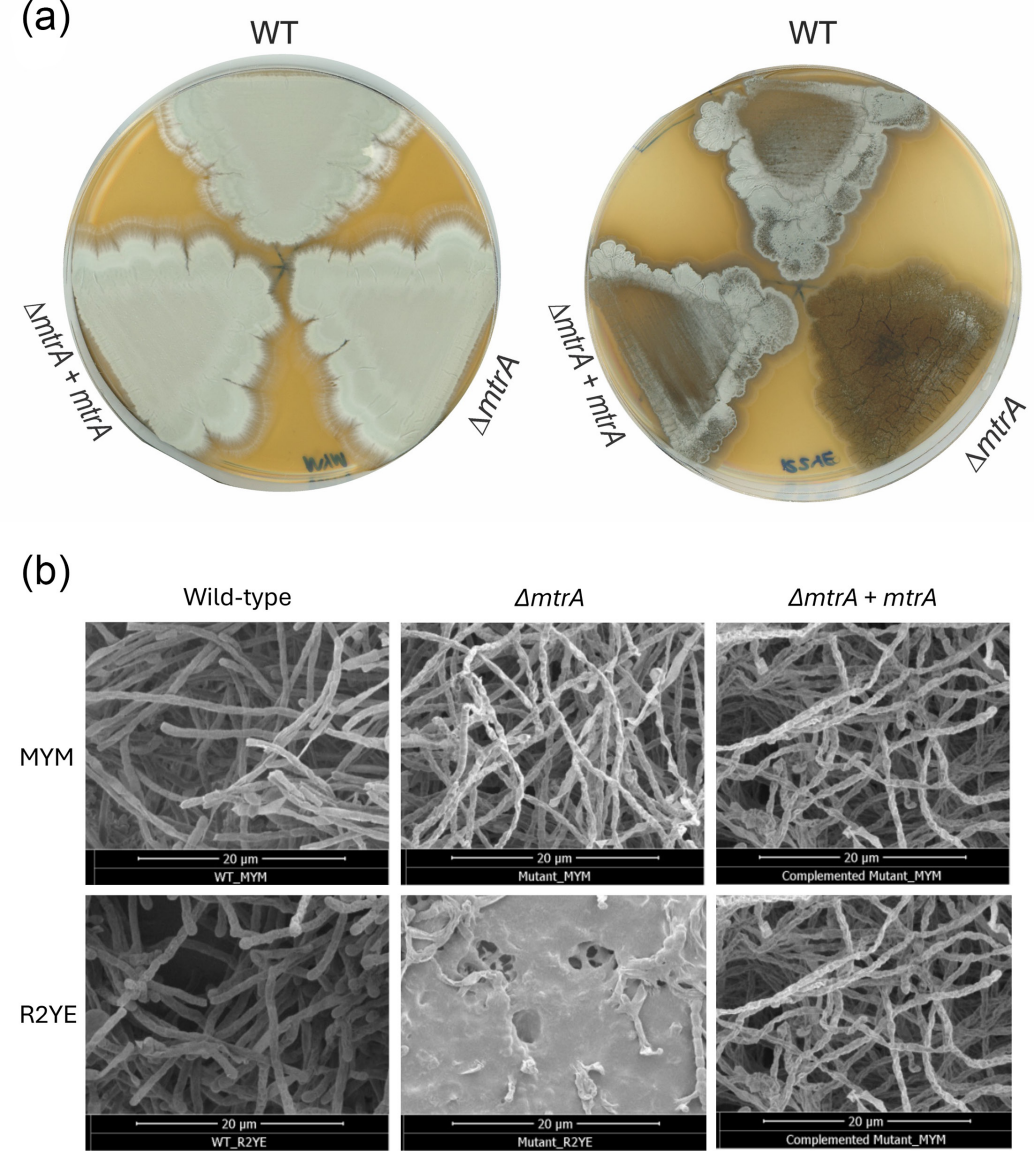
*S. venezuelae* ∆*mtrA* is conditionally bald and cannot sporulate on R2YE agar. Top. Seven-day-old cultures of S. venezuelae wild-type (WT), ∆*mtrA* and the ∆*mtrA* strain complemented in trans, grown on MYM agar (left) and R2YE agar without glucose (right). Substrate mycelium is brown, aerial mycelium is white and the spore chains are green due to the production of a green spore pigment. Bottom. Scanning electron micrographs of the same strains grown under the same conditions show that all strains are producing aerial hyphae and spores except for the ∆*mtrA* mutant growing on R2YE agar, where it only produces substrate hyphae with no aerial hyphae or spores.

Previous studies have shown that deletion of *mtrA* results in a conditional bald phenotype in *S. coelicolor* M145, *Streptomyces lividans* 1326 and *S. venezuelae* ISP5230. This means the *∆mtrA* mutants can sporulate on some growth media but not others; for example, *∆mtrA* mutants of all three species fail to sporulate on R2YE and R5 agar [22,29]. To test whether *mtrA* deletion blocks sporulation in *S. venezuelae* NRRL B-65442, we deleted the *mtrA* gene using pCRISPomyces-2 [32] and screened the *∆mtrA* mutant for defects in growth and development on MYM agar, a sporulation medium and on R2YE agar. Glucose was omitted from the R2YE agar recipe because it prevents the wild-type strain from sporulating. The results show that the *∆mtrA* mutant sporulates normally on MYM agar but fails to develop aerial hyphae or spores on R2YE agar, indicating that *S. venezuelae* NRRL B-65442 *∆mtrA* is conditionally bald (Fig. 1a). This was confirmed using scanning electron microscopy (Fig. 1b).

### Identification of MtrA binding sites upstream of genes required for hyphal growth, DNA replication and sporulation

We analysed existing *S. venezuelae* MtrA ChIP-seq datasets [1] to identify developmental genes with ChIP-seq peaks in their promoters or coding sequences, which indicate MtrA binding *in vivo*. MtrA binding was detected upstream of the developmental genes *adpA*, *bldM*, *filP*, *mtrA*, *ssgB* and *whiI*, which are required for aerial hyphae formation and sporulation and upstream of the *dnaA* gene which is essential for DNA replication (Fig. S1). These genes were chosen for further investigation because MtrA-dependent changes in their expression could be responsible for the conditional bald phenotype of the *S. venezuelae ∆mtrA* mutant. To pinpoint the precise MtrA binding motifs at these promoters, we used ReDCaT SPR. This technique involves designing double-stranded oligonucleotide probes that tile across a target promoter to narrow down the MtrA binding site [35]. Truncation and mutation of positive probes can then be used to identify the DNA sequence that is absolutely required for MtrA binding. The results show that all of these promoters contain recognizable MtrA-binding motifs that are bound by purified MtrA *in vitro*, with some promoters (*adpA*, *bldM* and *dnaA*) containing two or more MtrA binding motifs (Table 5, Fig. S2). MtrA also binds to the *mtrAB-lpqB* operon promoter, with the MtrA binding site positioned at −34 relative to the transcription start site, suggesting that MtrA activates the expression of *mtrAB-lpqB*. The *mtrA* gene is missing from the *∆mtrA* mutant, but the *mtrB* and *lpqB* gene products are reduced three- to fourfold in the absence of MtrA relative to the wild-type, which is consistent with MtrA-dependent activation of *mtrAB-lpqB* expression. However, the genes in this operon overlap, so we cannot rule out the possibility of polar effects on the downstream *mtrB-lpqB* genes due to *mtrA* deletion (Table 5). Previously identified MtrA-binding sites in *Streptomyces* and *Mycobacterium* species consist of two imperfect repeats of this motif separated by 6 nt, which are likely bound by dimeric MtrA. This suggests that in SPR, dimeric MtrA can bind to DNA using only a half site, i.e. a single motif. Zhang *et al*. used EMSAs to define the *Streptomyces* MtrA-binding site as a 17-bp GtnAcC-n5-GTnAcn sequence with a direct repeat of two motifs with a 5-bp spacer, whereas in *Mycobacterium* species, the MtrA-binding site is a direct repeat of the GTCACAgcg sequence motif [18,22].

**Table 5.** MtrA target promoters upstream of *S. venezuelae* developmental genes The MtrA-binding sites were identified using SPR and positions are given relative to the transcript start sites (TSS). Note that these appear to be MtrA half sites and that dimeric MtrA typically binds to a direct repeat of this sequence motif separated by 5 or 6 nt. TMT proteomics was used to measure the levels of each gene product after growth on either MYM or R2YE agar. Note that MtrA is missing from the *∆mtrA* mutant. MtrB and LpqB are encoded within the same operon as MtrA and are under the control of the *mtrA* promoter. Genes can be viewed using the StrepDB genome browser at http://streptomyces.org.uk, select ‘vnz chr’ from the drop-down Org and Mol list and search by gene number (e.g. vnz_12630). In all cases, an *∆mtrA* / WT abundance ratio of >2.0 or <0.7 has a *P*-value of <0.05. All others represent no significant change between *∆mtrA* and WT.

Gene name	Locus tag	Function	MtrA-binding sites	MtrA binding relative to TSS	TSS relative to ATG	Abundance ratio *∆mtrA*/WT	
						MYM	R2YE
*adpA*	vnz_12630	Development	TATCAAT TGACAGG	−325 −285	−96	0.998	0.981
*bldM*	vnz_22005	Development Orphan RR forms heterodimers with WhiI.	TGTCGAA TGTCTAA TTGCGAA TGCCGAA TCGCACA	+210 +105 −15 −42 −122	−127 (P1) −175 (P2) −255 (P3) −308 (P4)	1.685	0.278
*dnaA*	vnz_17980	DNA replication	TGGCGGG TTGCCCA TTACGGT	−259 −169 −122	−110	1.363	1.161
*filP*	vnz_24950	Development	TGCCCGT	−43	−39	0.958	0.783
*mtrA*	vnz_13525	RR, absent	TCTCAAG	−34	+1	0.003	0.001
*mtrB*	vnz_13520	SK	TCTCAAG	−34	–	0.268	0.221
*lpqB*	vnz_13515	Lipoprotein	TCTCAAG	−34	–	0.385	0.231
*ssgB*	vnz_05545	Cell division	TCACAAA	−55	−49	1.587	0.764
*whiD* (divergent from *bldM*)	vnz_22000	Spore maturation	TGTCGAA TGTCTAA TTGCGAA TGCCGAA TCGCACA		−188	nd	nd
*whiI*	vnz_28820	Orphan RR, Development	TCACCAA	−55	−82	2.611	0.152
**Indirect targets**							
*smeA*	vnz_04885	Class II BldM-WhiI target	n/a	n/a	n/a	nd	nd
*sffA*	vnz_04890 (in an operon with *smeA*)	Class II BldM-WhiI target	n/a	n/a	n/a	2.745	0.422
*ssgR*	vnz_18200	Class I BldM target	n/a	n/a	n/a	0.738	0.257
*ssgA*	vnz_05545	Controlled via SsgR	n/a	n/a	n/a	1.587	0.762
*whiB*	vnz_13645	Class I BldM target	n/a	n/a	n/a	1.757	0.549
*whiE locus*	vnz_33520, vnz_33515, vnz_33510, vnz_33505, vnz_33500	Class II BldM-WhiI target	n/a	n/a	n/a	3.506, 4.364, 2.697, 2.354, 2.609	0.663 0.346 0.872 1.093 0.378

### Defining the MtrA consensus binding sequence

**Fig. 2.**
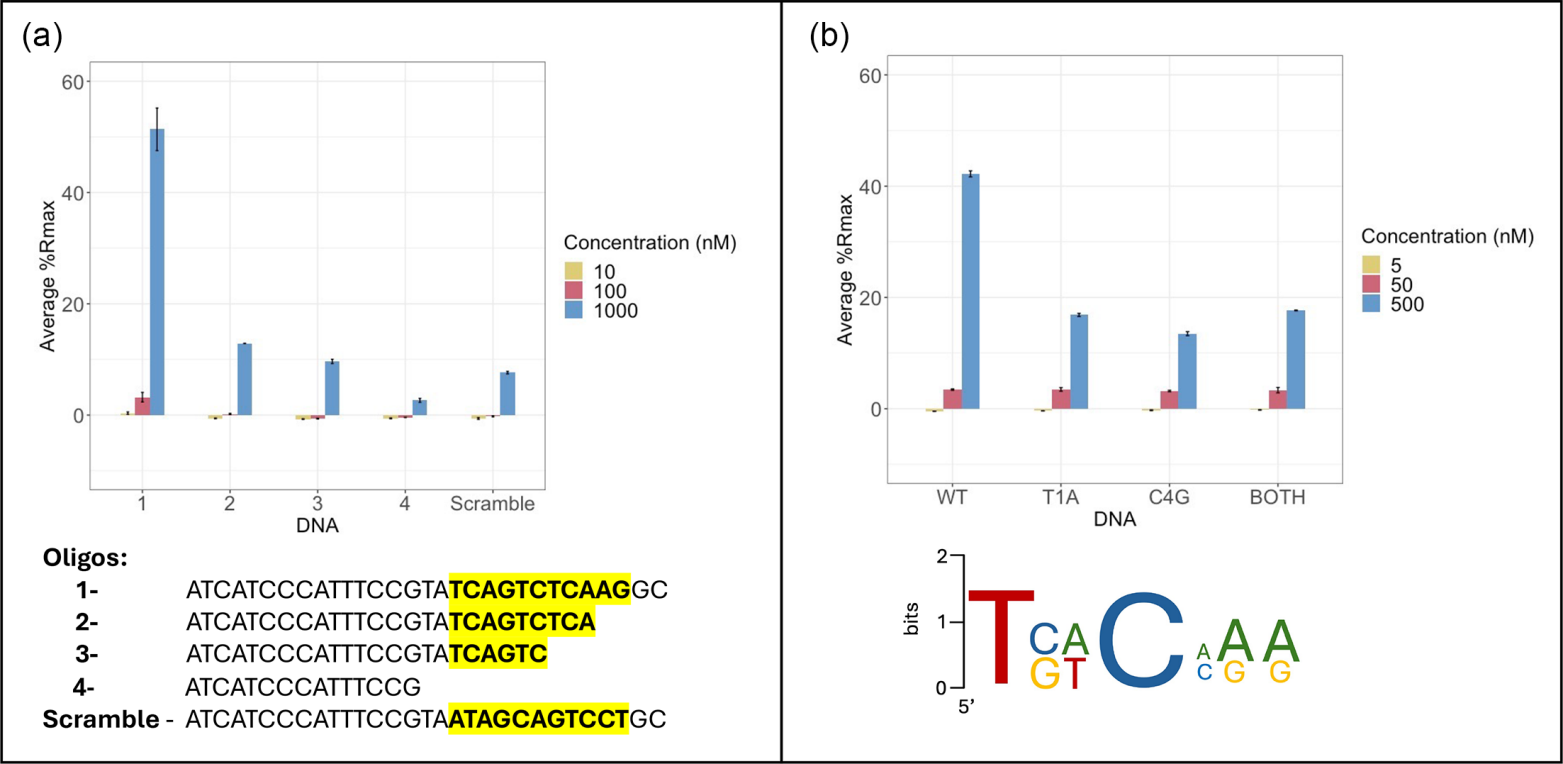
Consensus MtrA-binding site. (a) Alignment of the probes bound by MtrA *in vitro* revealed a 7-bp consensus sequence, which is likely a half-site. Removal of this motif (highlighted) by sequential truncation of the *mtrA* promoter probe abolished DNA binding. Similarly, scrambling the 7-bp binding motif within this oligo also significantly reduces DNA binding by purified MtrA.** (b)** Mutating either of the two most conserved nucleotides within the sequence of oligo 1 (T_1_ or C_4_) significantly reduced MtrA binding to DNA.

Alignment of the 14 experimentally verified MtrA-binding motifs shown in Table 5 results in a 7-base pair consensus sequence (Fig. 2). This 7-base pair consensus site sits at the 3′ edge of the *mtrA* promoter probe, so we sequentially truncated the probe until binding was abolished. Results show that any truncation of the 3′ edge of the probe affects the ability of MtrA to bind, and removal of the 15 bp containing the identified consensus sequence abolishes binding. To further confirm this sequence in the *mtrA* promoter as the MtrA-binding site, the 7-bp sequence was scrambled within the DNA probe, and this significantly reduced the ability of MtrA to bind (Fig. 2a). Notably, only two nucleotides in the consensus DNA-binding site, T at position 1 and C at position 4, are conserved across all identified hits, and mutation of either of these significantly reduced MtrA binding (Fig. 2b).

### D53 is a key residue for MtrA DNA binding

**Fig. 3.**
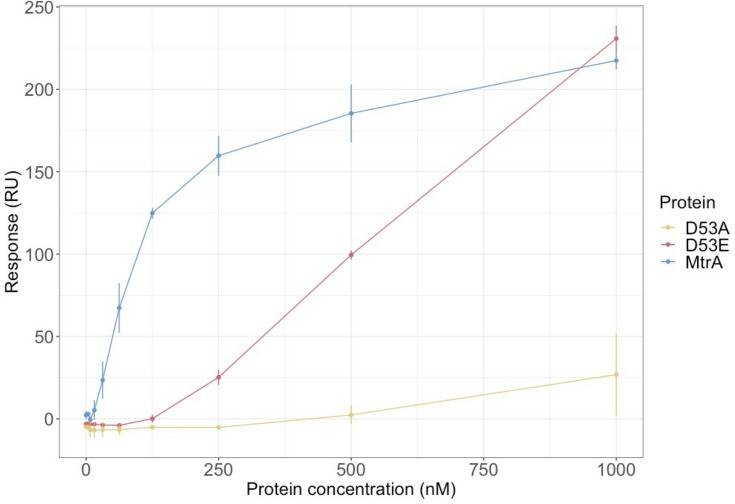
DNA binding by MtrA requires D53. ReDCaT SPR using the binding site identified at the *mtrA* promoter shows that the MtrA D53E variant has reduced DNA binding activity relative to the wild-type MtrA protein, suggesting this ‘phosphomimetic’ change only partially activates the protein. The D53A MtrA protein has no DNA-binding activity.

Upon activation by an environmental signal, MtrB is predicted to autophosphorylate using ATP and then phosphorylate MtrA on residue D53 [36]. In some (but not all) RRs, changing this aspartate to glutamate (D53E) can mimic phosphorylation and activate the protein, whereas changing the aspartate to alanine (D53A) inactivates typical RRs by preventing phosphorylation [37]. To investigate the binding of MtrA to DNA and its dependence on phosphorylation, the wild-type, D53A and D53E MtrA proteins were over-produced and purified from *E. coli*. Using the *mtrA* promoter probe, the DNA-binding affinities of the freshly purified proteins were measured *in vitro* using ReDCaT SPR. This revealed that wild-type MtrA has the strongest binding, but D53E MtrA retains some DNA binding activity to the *mtrA* promoter probe at higher concentrations. The D53A MtrA variant did not bind to this DNA probe (Fig. 3).

A recent study using *S. venezuelae* ATCC-10712 reported that rather than being phosphomimetic, a D53E change prevents MtrA from binding to DNA in electrophoretic mobility shift assays (EMSAs) and that complementing an *∆mtrA* mutant in trans with alleles encoding Flag-tagged D53A, D53E and D53N variants failed to pull down known MtrA target promoters in a ChIP-PCR experiment [36]. The divergence in our results may be because they used different promoters or because ReDCaT SPR is a more sensitive way of measuring *in vitro* DNA binding activity than EMSAs. We conclude from our data that D53 is the likely site of phosphorylation in *S. venezuelae* NRRL B-65442 MtrA and that the D53E MtrA variant retains some DNA binding activity, at least *in vitro*.

### MtrA directly controls the expression of key developmental regulatory genes

**Fig. 4.**
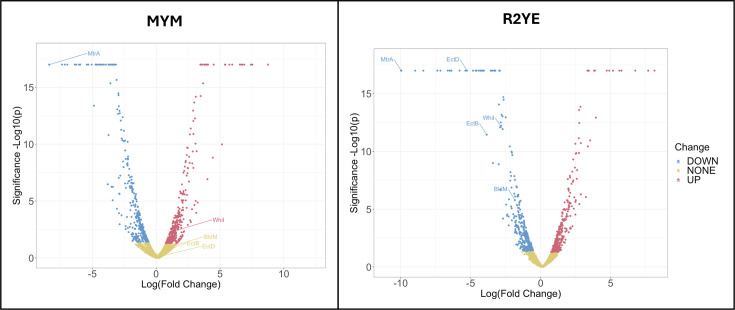
Loss of MtrA results in significantly lower levels of BldM and WhiI on R2YE agar. The volcano plot shows proteins whose levels are significantly differentially abundant (red or blue) in the *∆mtrA* mutant relative to wild-type following growth on MYM agar or R2YE agar. TMT proteomics of *S. venezuelae* shows that, when grown on R2YE agar, the ∆*mtrA* mutant has significantly lower levels of the developmental regulators BldM and WhiI and the ectoine biosynthetic enzymes EctB and EctD (highlighted with arrows on the volcano plot).

Next, we used TMT proteomics to examine the effects of *mtrA* deletion on the expression of developmental genes when the wild-type and *∆mtrA* strains were grown on R2YE and MYM agar (Fig. 4, Tables 5 and S3). Despite having experimentally verified MtrA binding sites, the expression of the *adpA*, *dnaA*, *ssgB* and *filP* genes was not significantly affected by loss of MtrA on MYM or R2YE agar. However, the expression of *bldM* and *whiI* was significantly affected on R2YE but not MYM agar, with BldM levels fourfold lower and WhiI levels sevenfold lower in the ∆*mtrA* mutant grown on R2YE agar (Table 5, Fig. 4). This suggests that MtrA directly activates the expression of both *bldM* and *whiI* on R2YE agar and that loss of MtrA leads to a reduction of BldM and WhiI that could be responsible for the bald phenotype of the *∆mtrA* strain (Fig. 1).

BldM and WhiI are key regulators of development and control the expression of genes required for aerial hyphae formation and sporulation (Table 5) [30]. As such, a *bldM* mutant is bald because it does not produce aerial hyphae, and a *whiI* mutant is white because it does not produce spores and therefore lacks the green WhiE spore-pigment. They are both atypical response regulators that do not require phosphorylation to bind to DNA. Homodimeric BldM regulates class I BldM target genes, which are required for the production of aerial hyphae, while heterodimeric BldM-WhiI regulates class II BldM target genes that are required for sporulation [30]. BldM activates the expression of *ssgR*, which encodes a transcription factor that in turn activates the expression of *ssgA*, which is required to correctly position Z rings prior to cell division (sporulation) [30]. Consistent with this, SsgR levels are fourfold lower in the *∆mtrA* mutant growing on R2YE agar, and SsgA levels are twofold lower, but their levels are not significantly affected on MYM agar (Table 5). Homodimeric BldM also activates *whiB* expression, which is twofold lower in the *∆mtrA* mutant grown on R2YE, but not MYM agar (Table 5). BldM-WhiI targets include the *whiE* operon [30] whose gene products make the green spore-pigment and are down-regulated up to threefold in the *∆mtrA* mutant grown on R2YE, but not MYM agar (Table 5). Thus, the combined TMT proteomics, ChIP-seq and ReDCaT SPR data are consistent with MtrA directly activating the production of BldM and WhiI and the expression of their downstream class I and class II target genes on R2YE agar. We hypothesize that loss of MtrA reduces the levels of BldM and WhiI under these growth conditions and results in the observed *∆mtrA* bald phenotype.

### Evidence that MtrAB senses and responds to osmotic stress

**Fig. 5.**
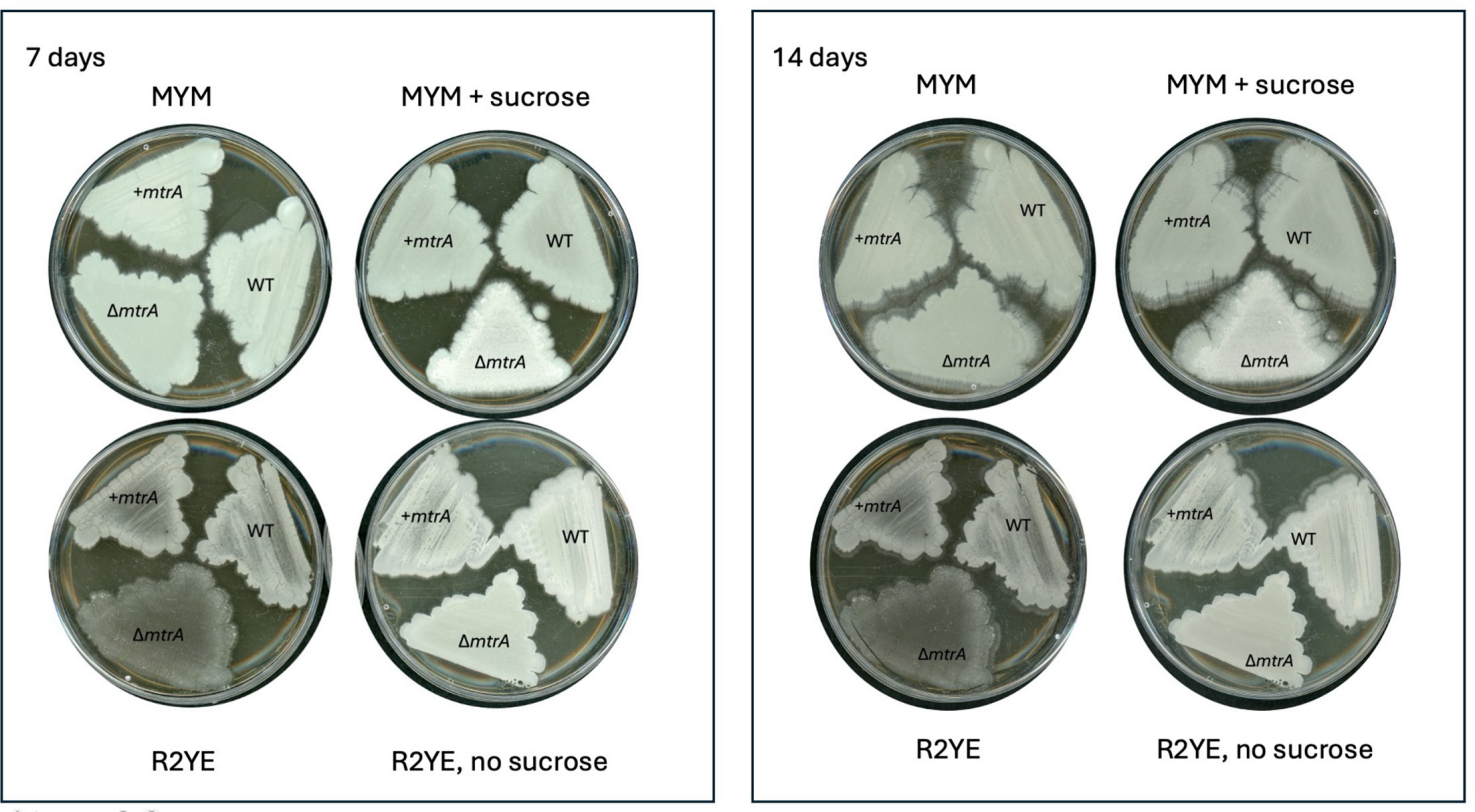
Sucrose induces the *∆mtrA* bald phenotype. *S. venezuelae* wild-type, *∆mtrA* and *∆mtrA+mtrA* (complemented) strains grown on MYM and R2YE agar with and without 10.2% sucrose for 7 and 14 days. The addition of sucrose to R2YE results in a bald *∆mtrA* phenotype, suggesting no aerial hyphae or spores, whereas in MYM plus sucrose, the *∆mtrA* mutant is white, suggesting it produces aerial hyphae but no spores.

The conditional bald phenotype of *∆mtrA* mutants in *Streptomyces* species suggests that MtrA is only required to activate entry into sporulation under certain growth conditions. The reported *Streptomyces ∆mtrA* mutants are all bald on R2YE agar, and notably, R2YE contains 10.2% sucrose, which likely induces osmotic stress. To test whether sucrose is responsible for the bald phenotype, we grew the wild-type, *∆mtrA*, and complemented *∆mtrA* strains on R2YE and MYM agar with and without 10.2% sucrose. The *∆mtrA* mutant is bald on R2YE agar and white on MYM agar containing 10.2% sucrose (Fig. 5), suggesting blocks at different stages of development depending on the growth medium, but neither strain forms mature green spores.

**Fig. 6.**
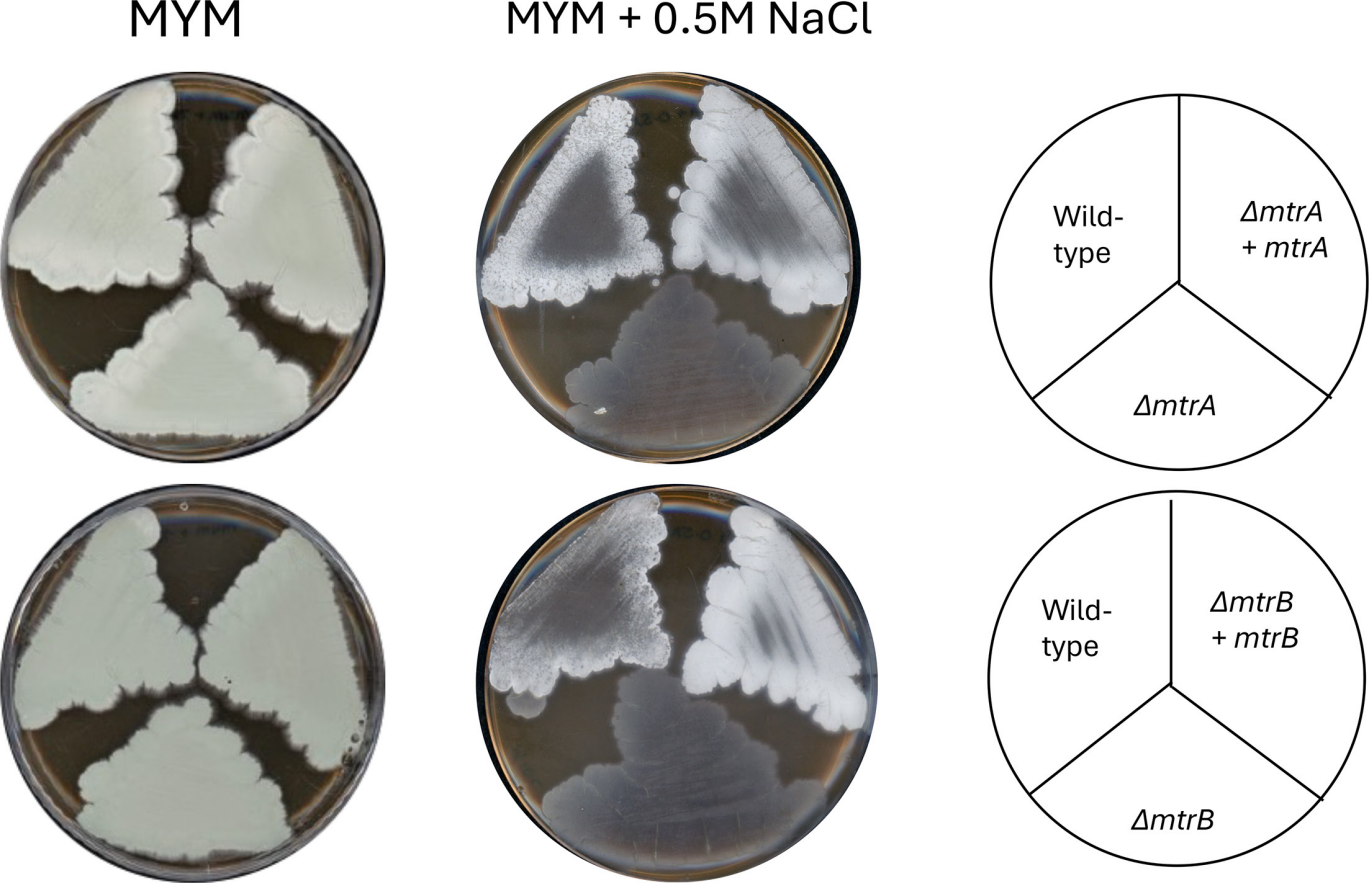
High salt induces a bald phenotype in the *∆mtrA* and *∆mtrB* strains. Cultures were grown at 30 °C for 7 days on MYM agar or MYM agar+0.5 M NaCl, as indicated. All the strains are sporulating on MYM agar, as indicated by the green spores. The wild-type and *in trans *complemented strains are forming white aerial mycelium, but no spores on MYM+0.5 M NaCl, but the *∆mtrA* and *∆mtrB* mutants are both bald.

*C. glutamicum* MtrB senses osmotic stress via its cytoplasmic domain and activates MtrA, which in turn activates the expression of *betP* and *proP*, which encode transporters for the compatible solutes betaine and proline [38,39]. Given that MtrAB forms a cognate two-component system and *S. venezuelae* MtrA activates the expression of *bldM* and *whiI* in response to high sucrose concentrations, it seems likely that *S. venezuelae* MtrB is also an osmotic stress sensor. To test this and our hypothesis that osmotic stress is causing the *∆mtrA* phenotype on MYM+sucrose, we plated *S. venezuelae ∆mtrA* and *∆mtrB* mutants on MYM agar with and without 0.5 M NaCl alongside the wild-type and *in trans* complemented mutant strains. High salt or sucrose concentrations induce hyperosmotic stress. The results show that the *∆mtrA* and *∆mtrB* mutants are both bald on MYM+0.5 M NaCl but develop normally on MYM agar (Fig. 6). The wild-type and complemented strains are delayed in growth and development on 0.5 M NaCl but are clearly forming white aerial mycelium. These observations support the hypothesis that MtrAB are both required to sense and respond to hyperosmotic stress and that MtrB activates MtrA.

**Fig. 7.**
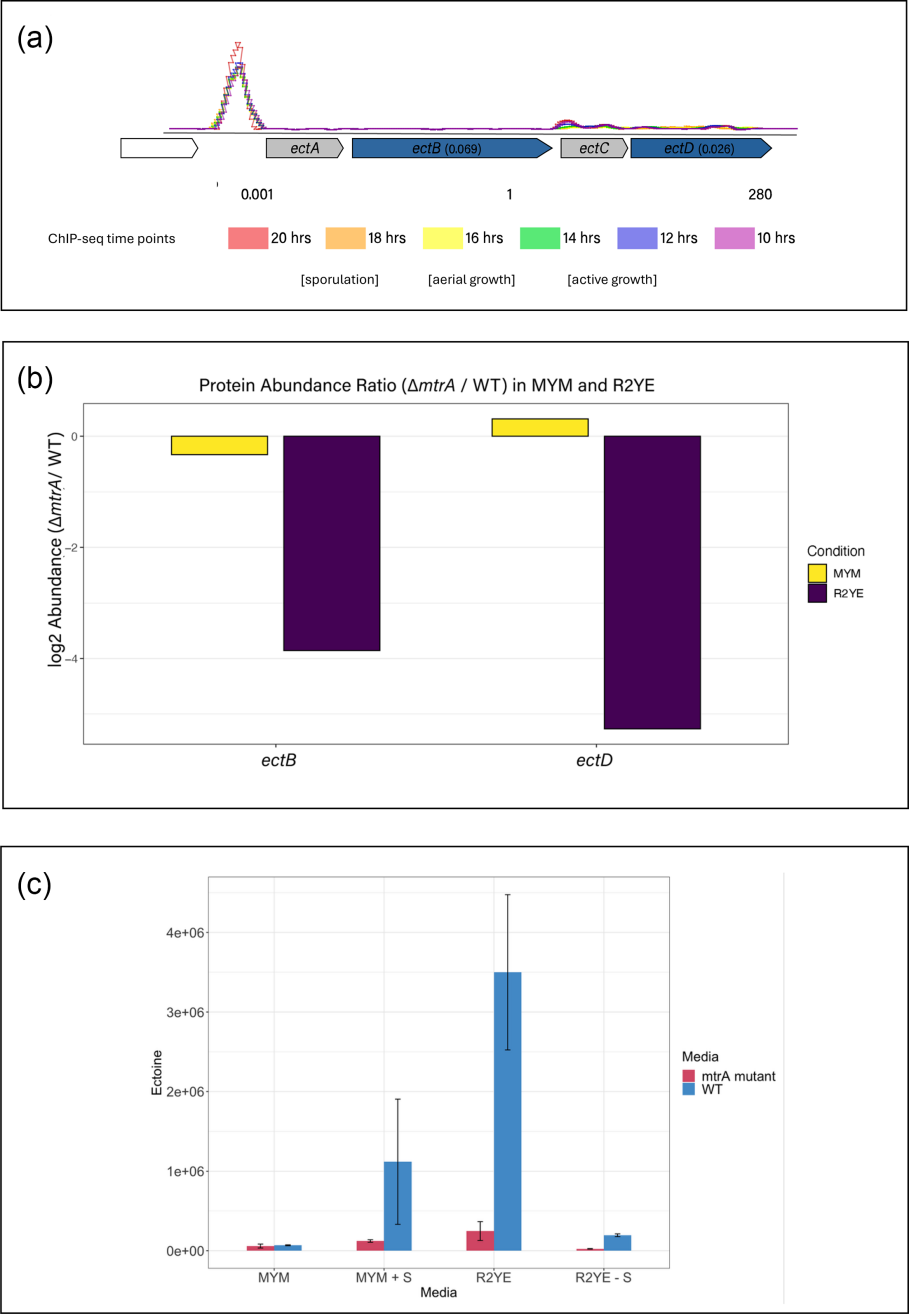
MtrA directly activates ectoine biosynthesis. (**a**). ChIP-seq data showing that MtrA binds to the *ectABCD* operon promoter throughout a developmental time course (time points are 10, 12, 14, 16, 18 and 20 h in liquid MYM medium [1]. (**b**) TMT proteomics data showing that the operon products EctB and EctD are 14- and 38-fold lower in the *∆mtrA* mutant relative to wild-type (WT), suggesting MtrA directly activates the expression of the *ectABCD* operon. EctA and EctC were not detected in this experiment. (**c**) LCMS data showing the levels of ectoine produced in *S. venezuelae* WT and ∆*mtrA* strains grown on MYM and R2YE agar with and without 10.2% sucrose (+S or –S). Ectoine production is activated in the presence of sucrose, and this is dependent on MtrA.

We also noticed that the *ectABCD* operon promoter is highly enriched in the MtrA ChIP-seq dataset throughout the developmental time course. In fact, this is the only promoter that was bound by MtrA at all time points throughout development (Fig. 7a). This operon encodes the biosynthetic pathway for the compatible solute and osmoprotectant ectoine, and we hypothesise that MtrA activates the expression of *ectABCD* in response to hyperosmotic stress. Consistent with this, the TMT data show that EctB and EctD are downregulated 14- and 38-fold, respectively, in the *∆mtrA* mutant relative to wild-type on R2YE agar (Fig. 7b, Table S3). The levels of these proteins were not significantly affected in the *∆mtrA* mutant grown on MYM agar (0.797 and 1.246, respectively). EctA and EctC were not detected in the TMT experiment. To test whether ectoine is produced in response to osmotic stress, we extracted and measured ectoine levels in the *S. venezuelae* wild-type and *∆mtrA* mutant grown on MYM or R2YE agar with and without sucrose. The results show a significant increase in ectoine production in the wild-type strain in the presence of 10.2% sucrose, but this is abolished in the *∆mtrA* mutant (Fig. 7c). Taken together, the data presented in Fig. 7 indicate that MtrA directly activates ectoine biosynthesis when *S. venezuelae* is grown on 10.2% sucrose. Given that ectoine is produced to protect *Streptomyces* bacteria against heat and osmotic stress, we propose that MtrAB activates ectoine biosynthesis as a direct response to hyperosmotic stress.

It is important to note that the ChIP-seq experiment was performed on cultures of *S. venezuelae* grown in liquid MYM medium with no sucrose. This suggests that MtrA binds to the *ectA* promoter in the absence of sucrose but only activates *ectABCD* expression when sucrose is added to the growth medium. This is consistent with our observation that MtrA represses chloramphenicol biosynthesis in MYM-grown cultures. If MtrA binds to DNA targets in the absence of phosphorylation, it would still repress target genes by occluding RNA polymerase access to the target gene promoter, but phosphorylation would be required to activate target gene expression. The binding and repression of genes by unphosphorylated response regulators is not unprecedented. In *Salmonella enterica*, the response regulator SsrB also binds to target DNA in the absence of SsrA (its cognate sensor kinase), and SsrB activates expression of *csgD* in an *∆ssrA* mutant by disrupting H-NS-mediated silencing of the DNA. However, for direct RNA polymerase-mediated activation of its SPI-2 target genes, SsrB requires phosphorylation by SsrA [40]. In *E. coli*, the well-characterized two-component system EnvZ-OmpR has also been shown to undertake canonical (phosphorelay) and non-canonical signalling, where the latter does not require phosphorylation of OmpR for it to activate or repress target genes. Under certain growth conditions, e.g. low pH, OmpR interacts with EnvZ and dimerizes but no phosphorylation occurs. This dimerization is still sufficient for DNA binding by OmpR and activation or repression of target genes [41].

### Over-expression of *bldM* and *whiI* restores normal development in the *∆mtrA* mutant

**Fig. 8.**
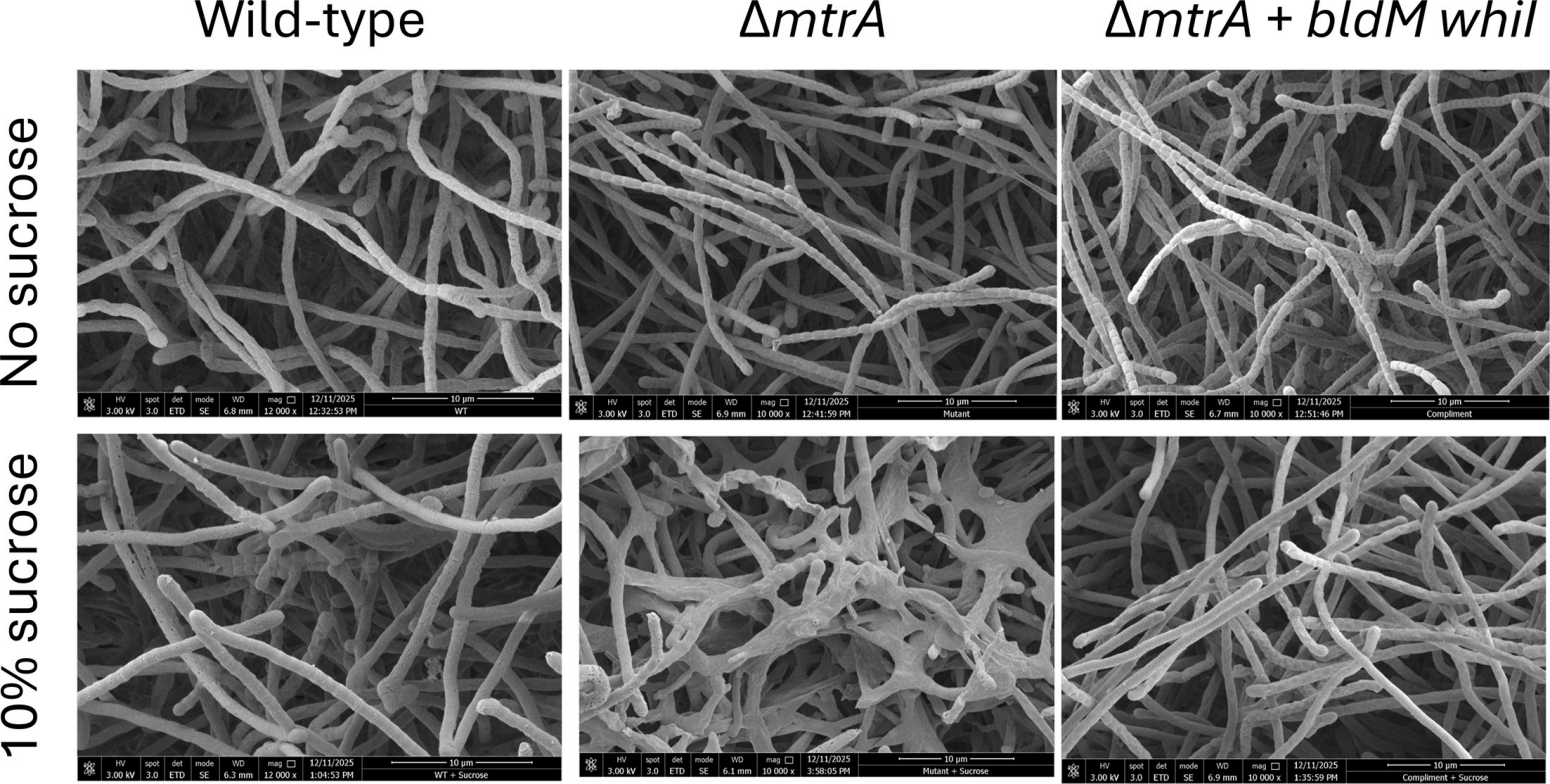
Over-expression of *bldM* and *whiI* in the *∆mtrA* mutant. Scanning electron micrographs were taken after 10 days of growth on MYM agar with or without 10.2% sucrose, as indicated. The images show that even after this extended incubation, *S. venezuelae ∆mtrA* is unable to develop normal spore chains in the presence of 10% sucrose. However, artificial over-expression of *bldM* and *whiI* in a synthetic operon under the control of the constitutive high-level ermE* promoter restores sporulation to the *∆mtrA* mutant grown in the presence of sucrose.

To test whether the reduced levels of BldM and WhiI are responsible for the conditional bald phenotype of the *∆mtrA* mutant during growth on high sucrose, we over-expressed the *bldM* and *whiI* genes in the *∆mtrA* mutant using the constitutive high-level ermE* promoter. These strains were then grown on MYM agar with and without 10.2% sucrose. The results show that in the presence of sucrose, the *mtrA* mutant produces aerial hyphae, as suggested by the white mycelium of plate-grown cultures (Fig. 5) but is unable to sporulate. However, sporulation is restored by over-expressing *bldM* and *whiI* in the *∆mtrA* strain growing on MYM with 10.2% sucrose (Fig. 8). This supports our hypothesis that MtrAB is required to activate the expression of *bldM* and *whiI* in the presence of 10.2% sucrose and trigger sporulation. Deletion of *mtrA* leads to a reduction in BldM and WhiI levels under these growth conditions and results in the (conditional) bald phenotype.

This work further illustrates the complexity of *Streptomyces* life cycle regulation, where sporulation must be controlled temporally and in response to both biotic and abiotic stress. BldM and WhiI act together to initiate sporulation [30], and both are under the control of BldD, the master repressor of sporulation [42]. BldD directly represses the expression of *bldM* and *whiG*, which encodes the RNA polymerase sigma factor σ^WhiG^ and RNA polymerase containing σ^WhiG^ directs transcription of *whiI* [10]. σ^WhiG^ activity is also controlled post-translationally, by the anti-σ factor RsiG [43], and the activities of BldD and RsiG are controlled by binding of the secondary messenger cyclic-di-GMP [10,44]. Thus, a reduction in intracellular c-di-GMP levels (in response to unknown signals) relieves BldD repression of *bldM* and *whiG* and also removes RsiG inhibition of σ^WhiG^, leading to σ^WhiG^-directed transcription of *whiI* [45].

**Fig. 9.**
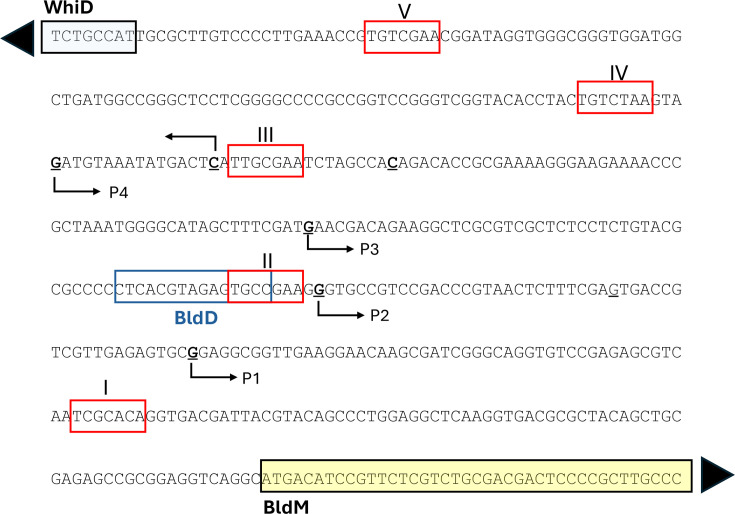
The divergent *whiD-bldM* promoters in *S. venezuelae*. The *whiD* and *bldM* developmental genes are divergently transcribed, and their back-to-back promoters contain five MtrA-binding sites, marked with red boxes and numbered V-I. The BldD-binding site (blue box) overlaps the MtrA-binding site II. There are four putative *bldM* transcript start sites, marked with arrows, and the major transcript initiates from P1 (see also Fig. S3). The *whiD* gene has a single transcript start site, and expression of this gene is likely inhibited by MtrA binding to sites V, IV and III. The *whiD* coding sequence is shaded light blue, and the *bldM* coding sequence is shaded yellow.

It is not clear how MtrA bypasses BldD and RsiG repression to simultaneously activate the expression of both *bldM* and *whiI* or if MtrA-dependent activation of *whiI* requires σ^WhiG^. The levels of BldD, σ^WhiG^ and RsiG were not significantly affected by the loss of MtrA on either MYM or R2YE agar in this work (Table S2). The ReDCaT SPR experiments show that purified MtrA binds to five different sites upstream of *bldM*, which is divergent from the *whiD* gene (Fig. 9). WhiD is required for spore maturation and belongs to the WhiB-like family of transcription factors, but its target genes are not known. MtrA binding to sites III, IV and V would likely repress expression of *whiD*, but the WhiD protein was not detected in the TMT proteomics experiment, so we do not know if it is regulated by MtrA (Table S5). The *bldM* gene potentially has four transcription start sites (TSS) (Figs 9 and S3), and if these represent true promoters, then some would be repressed by MtrA binding to sites I-IV, while others could be activated. Although all five sites were bound by MtrA *in vitro,* we do not know which are bound by MtrA *in vivo* and are physiologically relevant. MtrA binds to more than 1,000 sites across the genome during the life cycle of *S. venezuelae*, but it is unlikely that they all have a regulatory role. The WhiA transcription factor is similar in that it binds to many sites but requires interaction with WhiB to contact RNA polymerase and activate transcription [46,47]. Given that MtrA activates expression of *bldM,* it seems likely that MtrA binding to site II could be important in activating transcription from TSS P1. Intriguingly, the predicted BldD-binding site overlaps MtrA-binding site II, and BldD or MtrA binding at this site would repress transcription from the P4-P2 TSS. It is also possible that BldD represses P1 by blocking MtrA binding to site II, but this needs to be tested. MtrA binding site I is downstream of all four TSS and positioned such that bound MtrA would block transcription of *bldM*. It will be necessary to mutate each of these sites individually to discover which are involved in the activation of *bldM*.

MtrA binds to only a single site at the *whiI* promoter, and this is positioned at −55 relative to the TSS, which is consistent with MtrA-dependent activation via contact with bound RNA polymerase. It will be important to understand which of the MtrA binding sites identified at the *bldM* promoter are actually bound by MtrA *in vivo* under osmotic stress conditions and if or how MtrA interacts with BldD and σ^WhiG^. The *bldD* promoter is directly repressed by MtrA in *S. avermitilis* and *S. coelicolor* [22,29], but *bldD* was not identified as an MtrA target in ChIP-seq experiments in *S. venezuelae* NRRL B-65442 [1], and the *bldD* promoter was not bound by purified MtrA in the ReDCaT SPR experiments (not shown).

## Concluding remarks

Here, we have shown that the conditional bald phenotype of *S. venezuelae ∆mtrA* is caused by the addition of 10.2% sucrose to the growth medium, and neither *S. venezuelae ∆mtrA* nor *∆mtrB* strains can form spores when grown in the presence of 0.5 M NaCl. We propose, therefore, that MtrAB is an osmotic stress-sensing TCS, whereby MtrB senses osmotic stress and MtrA responds by activating the production of the compatible solute ectoine and triggering entry into sporulation via activation of BldM and WhiI to increase the chances of survival [30]. MtrAB senses and responds to osmotic stress in *C. glutamicum* and *Dietzia* spp*.* and is responsive to changes in pH in mycobacteria. Given that pH changes are closely linked to osmotic stress, this suggests that the osmotic stress-sensing function of MtrAB is widely conserved in the phylum *Actinomycetota* [21,39, 48]. Detailed characterization of *C. glutamicum* MtrB (Cgl-MtrB) revealed that it senses osmotic stress via its cytoplasmic domain. However, MtrB also has a large extracellular sensor domain, and it has been proposed that it likely senses more than one signal [38]. This might explain why MtrAB has also been implicated in the regulation of carbon and nitrogen metabolism and phosphate uptake in *Streptomyces* species and in virulence and intracellular survival in pathogenic mycobacteria.

Our data also suggest that MtrA can bind to its DNA targets *in vivo* in the absence of hyperosmotic stress. ChIP-seq experiments were carried out in a previous study, in which *S. venezuelae* was grown in liquid MYM medium and sampled at 2 h time points throughout the life cycle [1]. This is because *S. venezuelae* sporulates in liquid MYM medium and completes its life cycle within 20 h and because at the time we did not know that MtrA could be induced by NaCl or sucrose. Despite the lack of osmolytes, MtrA still bound to >1,000 target promoters across the developmental time course. For example, MtrA was bound to the promoter of the *ectABCD* operon at all the time points tested through the life cycle, and yet, MtrA does not activate *ectABCD* or ectoine biosynthesis unless sucrose is present (Fig. 7). We propose that MtrA can bind to DNA in the absence of phosphorylation but requires phosphorylation to activate target gene expression. This would explain why it can repress target genes in MYM medium with no osmolyte and why deletion of *mtrA* activates chloramphenicol biosynthesis under these conditions. There are multiple examples in the literature of bacterial response regulators such as SsrA acting in this way, but typically they require dimerization via interaction with and/or phosphorylation by their cognate sensor kinases to activate target gene expression [40].

In future work, it will be important to understand how MtrAB fits into the bigger picture of *Streptomyces* development and the hyperosmotic stress response. These bacteria live in constantly fluctuating soil environments, where osmotic stress is likely to present a frequent danger and several osmotic stress-sensing signal transduction systems have been identified, including OsaAB. This TCS consists of a yeast-like hybrid sensor kinase called OsaA that controls the activity of a response regulator called OsaB and *S. coelicolor ∆osaB* is bald on R2YE agar. The regulon of genes under OsaB control is not known, but *S. coelicolor osaB* expression is induced by hyperosmotic stress in a σ^B^-dependent manner, as is the expression of *sigB* itself [49]. σ^B^ is a master regulator of the osmotic stress response in Gram-positive bacteria but remains poorly characterized in *Streptomyces* species [50]. OsaC is encoded divergently from the *osaAB* operon and appears to function as an anti-anti-σ factor that controls the activity of σ^B^. The secondary messenger c-di-AMP has also been implicated in the osmotic stress response in *S. venezuelae* and other Gram-positive bacteria. Deletion of *S. venezuelae disA*, which encodes a diadenylate cyclase, results in low levels of c-di-AMP and hypersensitivity to 0.5 M NaCl [51]. In this work, the levels of OsaAB were not significantly affected by loss of MtrA on either MYM or R2YE agar, and σ^B^ was not detected in the TMT proteomics, while DisA was only slightly less abundant in the *∆mtrA* mutant grown on R2YE agar (0.696) compared to wild-type *S. venezuelae* (Table S1). It will be important to identify target genes for OsaB and σ^B^, compare these to the MtrA regulon and examine their expression in the presence and absence of osmolytes in the same species.

## Supplementary material

10.1099/mic.0.001706 Uncited Supplementary Material 1.

10.1099/mic.0.001706 Uncited Supplementary Material 2.

## Abbreviations

cryo-SEM: cryo-scanning electron microscopy
EMSAs: electrophoretic mobility shift assays
LC-ESI-MS/MS: liquid chromatography coupled with electrospray ionization tandem mass spectrometry
PD: Proteome Discoverer
ReDCaT: reusable DNA capture technology
SPR: surface plasmon resonance
SPS: synchronous precursor selection
TCS: two-component signal transduction systems
TFA: trifluoroacetic acid
TMT: tandem mass tag
TSS: transcription start sites
