# Supplementary material for: MtrAB activates ectoine production and triggers sporulation in response to osmotic stress in Streptomyces venezuelae
Source: Microbiology (Reading). 2026 May 20;172(5):001706. doi: 10.1099/mic.0.001706 (PMC13189361; doi:10.1099/mic.0.001706)
Supplement: Uncited Supplementary Material 1. [file mic-172-01706-s001.pdf]

MtrAB activates ectoine production and triggers sporulation in response to osmotic stress in *Streptomyces venezuelae*

Supplementary Information

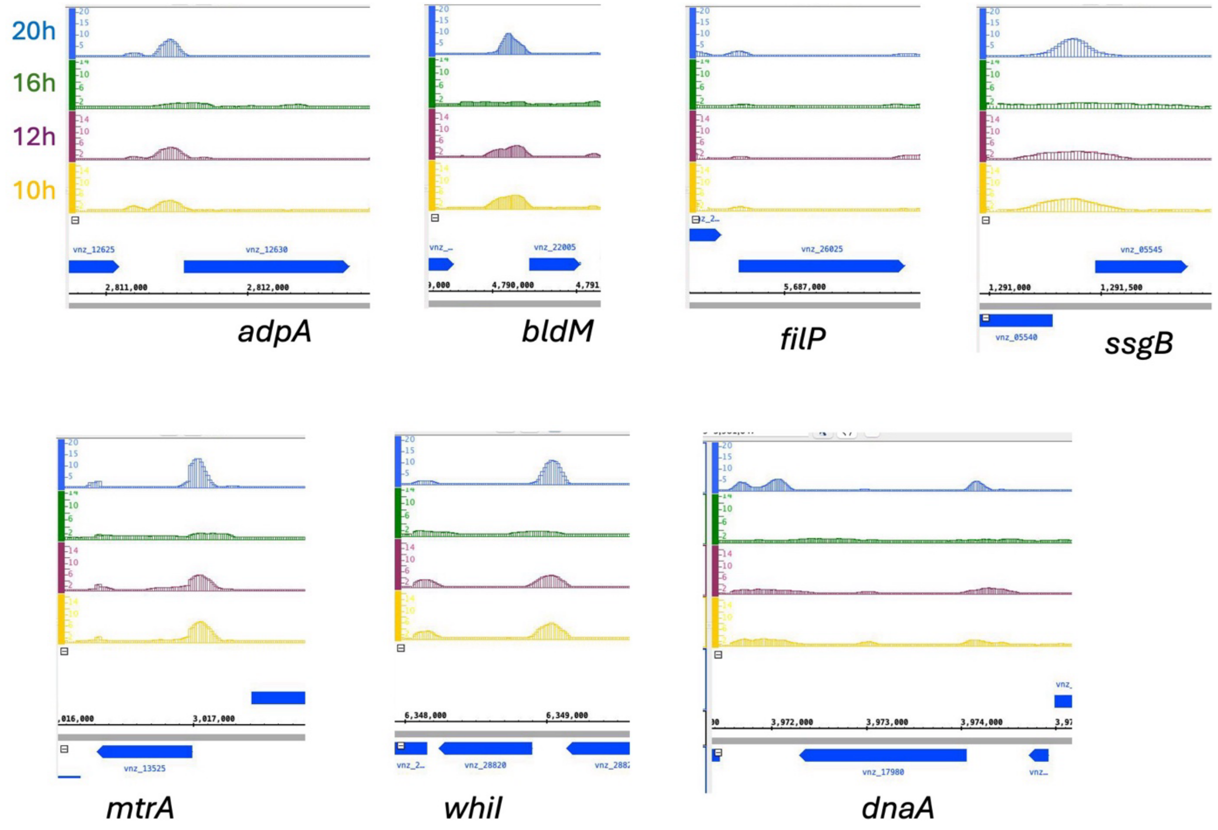

Figure S1. MtrA ChIP-Seq peaks for key developmental genes in *S. venezualae*. MtrA-FLAG binds promoters of key developmental genes in *S. venezuelae* (Som *et al.*, 2017). The colours represent different culturing times, 10, 12, 16 and 20 hours on liquid MYM medium, as indicated.

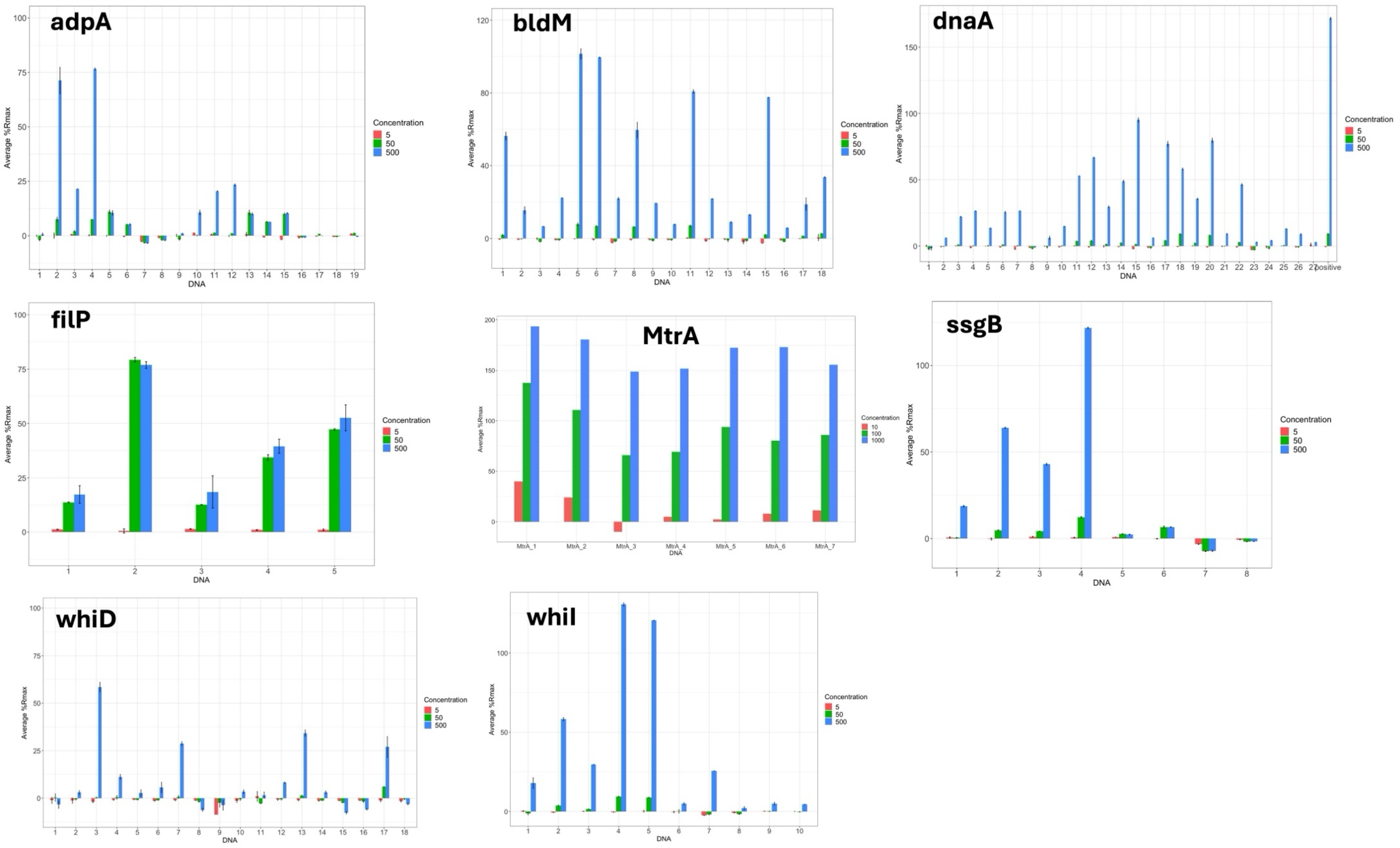
adpA
bldM
dnaA
filP
MtrA
ssgB
whiD
whiI
Average %Rmax
DNA
Concentration
5
50
500
10
100
1000
positive
MtrA_1
MtrA_2
MtrA_3
MtrA_4
MtrA_5
MtrA_6
MtrA_7

Figure S2. ReDCaT SPR data for MtrA. These *in vitro* DNA binding assays used double stranded oligonucleotide probes which tiled across promoters of interest to identify the exact MtrA binding sites. The chosen promoters were all enriched in the *S. venezuelae* MtrA ChIP-seq dataset and drive the expression of developmental genes, as shown. The x-axis shows the DNA probe number (the oligo sequences are shown in Table S2), and the y-axis shows the average Rmax which indicates the strength of binding of purified MtrA protein. The higher the bar, the stronger the binding by MtrA.

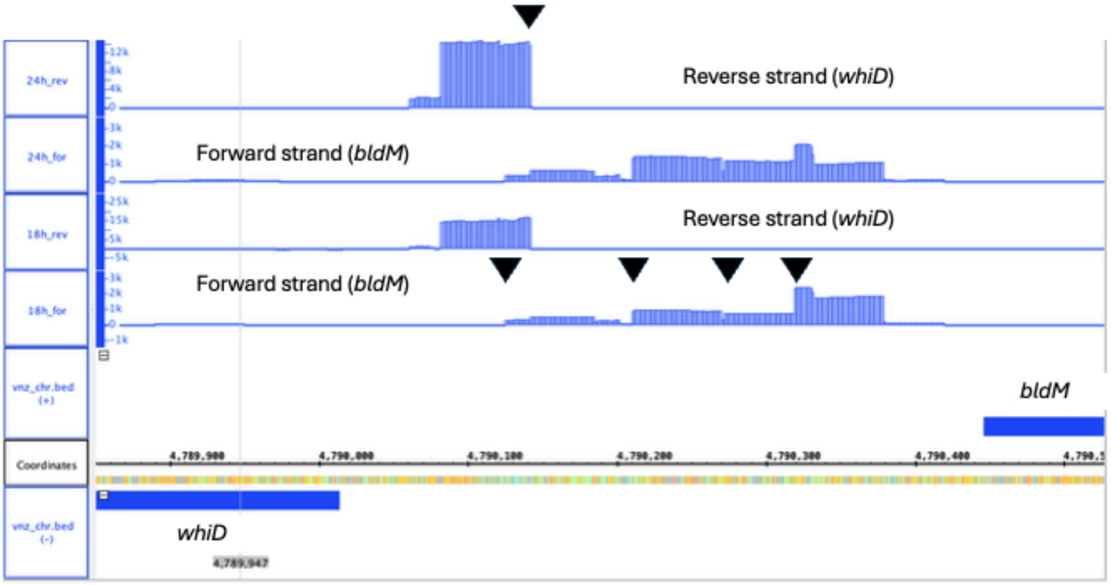

Figure S3. Differential RNA-seq data for the divergent *whiD-bldM* promoters. This shows the dRNA-seq data for 18h and 24h cultures of wild-type *S. venezuelae* in liquid MYM medium viewed in the Integrated Genome Browser (IGB). The data can be downloaded here: https://streptomyces.org.uk/vnz_tss.html with instructions on how to view it in IGB. The sequences are ~60bp so anything wider indicates additional transcript start sites (TSS). These are marked with arrows in the reverse strand sequencing (*whiD)* and forward strand sequencing (*bldM*).

Table S1. Primers used in this study

Name	Description	Sequence
NS-145	*mtrA* forward BamHI (pETduet)	GGATCCTAAAGGCCGCGTTCTGGTCGTC
NS-146	*mtrA* reverse HindIII (pETduet)	AAGCTT TCA GCTCGGTCCCGCCTTGTACC
NS301	*mtrA* forward HindIII (pSS170)	ccatcagcaaaaggggatgataagtttatcCCGTGGGCCGGTCCC
NS301	*mtrA* reverse HindIII (pSS170)	CGAATCGATGATCATATGAGAGAATCTtcagctcggtcccgcctt
NS304	*mtrA* left arm in pCRISP	gaacgctcggttgccgccgggcgtttttaGACCGGGCGGCCGAG
NS305	*mtrA* left arm in pCRISP	ccacgatctccgggcgctccgggtcATCGACATCATCCCATTTCCGTATC

NS306	*mtrA* right arm in pCRISP	AGACTGATACGGAAATGGGATGATGTCGATgacccggagcgcccg
NS307	*mtrA* right arm in pCRISP	ttttacggttcctggccgcgccggggccgCTCGCCGGTGACCTTCATCC
NS308	*mtrA* spacer 1 For	ACGCCGACCTGATGCTGCCCGGAA
NS309	*mtrA* spacer 1 Rev	AAACTTCCGGGCAGCATCAGGTCG
NS365	*pETduet D53 For GA*	ccacagccaggatccTAAAGGCCGCGTTCTGGTCGTCGACGACGACACCG
NS366	*pETduet D53 Rev GA*	TTAAGCATTATGCGGCCGCAAGCTTtcagctcggtcccgcctt
RD001	*bldM* for NdeI	gtctagaacaggaggccccatatgATGACATCCGTTCTCGTC
RD002	*bldM* rev plus RBS	TTACCTCCGATGTTGAGTGCGATTCAGCGAACCAG
RD003	*whiI* For plus RBS	CTCAACATCGGAGGTAAGCCATGTCCGTTCTCCTCGAGC
RD004	*whiI* Rev HindIII	ctcatgagaacctaggatccaagcttGTCCGTCAGTGGATGATC

Table S2. Oligonucleotides used for ReDCaT SPR. Upper case represents promoter sequence, lower case is the ReDCaT linker for annealing to the chip.

Primer name	Sequence
ssgBFor_1	GGTCCCAACTTACCCGACGGCGGGCAGTCGCCGGAATGGC
ssgBRev_1	GCCATTCCGGCGACTGCCCGCCGTCGGGTAAGTTGGGACCcctaccctacgtcctcctgc
ssgBFor_2	AGTCGCCGGAATGGCGGTATTACCGCAGGTCACACGCCCC
ssgBRev_2	GGGGCGTGTGACCTGCGGTAATACCGCCATTCCGGCGACTcctaccctacgtcctcctgc
ssgBFor_3	CAGGTCACACGCCCCCATACGAGCTACCTGAACGATCACA
ssgBRev_3	TGTGATCGTTCAGGTAGCTCGTATGGGGGCGTGTGACCTGcctaccctacgtcctcctgc
ssgBFor_4	ACCTGAACGATCACAAAACCGACAGAGGGGTTTACAACGG
ssgBRev_4	CCGTTGTAAACCCCTCTGTCGGTTTTGTGATCGTTCAGGTcctaccctacgtcctcctgc
ssgBFor_5	AGGGGTTTACAACGGCACTGGAGGTGGCATGTCGATTTCG
ssgBRev_5	CGAAATCGACATGCCACCTCCAGTGCCGTTGTAAACCCCTcctaccctacgtcctcctgc
ssgBFor_6	GGCATGTCGATTTCGCCGACGTGCGAATCCCCGAGCGCAC
ssgBRev_6	GTGCGCTCGGGGATTCGCACGTCGGCGAAATCGACATGCCcctaccctacgtcctcctgc
ssgBFor_7	AATCCCCGAGCGCACACTGAGCGAAAGGCCCTGGCGCTTA
ssgBRev_7	TAAGCGCCAGGGCCTTTCGCTCAGTGTGCGCTCGGGGATTcctaccctacgtcctcctgc
ssgBFor_8	AGGCCCTGGCGCTTATGAACACCAC
ssgBRev_8	GTGGTGTTCATAAGCGCCAGGGCCTcctaccctacgtcctcctgc
adpAFor_1	GGGCCCGGCCCACGGGGCGGACCCCGGGGGAGAAACCGGT
adpaARev_1	ACCGGTTTCTCCCCCGGGGTCCGCCCCGTGGGCCGGGCCCcctaccctacgtcctcctgc
adpAFor_1	GGGCCCGGCCCACGGGGCGGACCCCGGGGGAGAAACCGGT
adpARev_1	ACCGGTTTCTCCCCCGGGGTCCGCCCCGTGGGCCGGGCCCcctaccctacgtcctcctgc
adpAFor_2	GGGGGAGAAACCGGTATCAATCGGGGCGCCGACGGGGCCC
adpARev_2	GGGCCCCGTCGGCGCCCCGATTGATACCGGTTTCTCCCCCcctaccctacgtcctcctgc
adpAFor_3	GCGCCGACGGGGCCCGCTCCTCGTGGAGTTGACAGGTTCG
adpARev_3	CGAACCTGTCAACTCCACGAGGAGCGGGCCCCGTCGGCGCcctaccctacgtcctcctgc
adpAFor_4	GAGTTGACAGGTTCGTACACCCGAACCCATATGTTTTCAG
adpARev_4	CTGAAAACATATGGGTTCGGGTGTACGAACCTGTCAACTCcctaccctacgtcctcctgc
adpAFor_5	CCCATATGTTTTCAGCCAAGTTCCATCTGTCGGCGAGTCC
adpARev_5	GGACTCGCCGACAGATGGAACTTGGCTGAAAACATATGGGcctaccctacgtcctcctgc
adpAFor_6	TCTGTCGGCGAGTCCTTGGCGGAACGGTTGCCCAACCCCG
adpARev_6	CGGGGTTGGGCAACCGTTCCGCCAAGGACTCGCCGACAGAcctaccctacgtcctcctgc
adpAFor_7	GGTTGCCCAACCCCGGCGCCGGCACCCGAGAATGCCCCGC

adpARev_7	GCGGGGCATTCTCGGGTGCCGGCGCCGGGGTTGGGCAACCcctaccctacgtcctcctgc
adpAFor_8	CCGAGAATGCCCCGCCACAAGGGCCCGCACCCCCCGCGTA
adpARev_8	TACGCGGGGGGTGCGGGCCCTTGTGGCGGGGCATTCTCGGcctaccctacgtcctcctgc
adpAFor_9	CGCACCCCCCGCGTACGGGGGCGTTCGCCACGCCGCCCCT
adpARev_9	AGGGGCGGCGTGGCGAACGCCCCCGTACGCGGGGGGTGCGcctaccctacgtcctcctgc
adpAFor_10	CGCCACGCCGCCCCTTCCCCGGGCGGCCATCGGACGGAAG
adpARev_10	CTTCCGTCCGATGGCCGCCCGGGGAAGGGGCGGCGTGGCGcctaccctacgtcctcctgc
adpAFor_11	GCCATCGGACGGAAGACTTCGCGATCGATCGCTTCACGCC
adpARev_11	GGCGTGAAGCGATCGATCGCGAAGTCTTCCGTCCGATGGCcctaccctacgtcctcctgc
adpAFor_12	CGATCGCTTCACGCCAAGTGGCCTTGTCGACAATCCACCG
adpARev_12	CGGTGGATTGTCGACAAGGCCACTTGGCGTGAAGCGATCGcctaccctacgtcctcctgc
adpAFor_13	GTCGACAATCCACCGGATGGAGAACTTGTCACGCCGGCGG
adpARev_13	CCGCCGGCGTGACAAGTTCTCCATCCGGTGGATTGTCGACcctaccctacgtcctcctgc
adpAFor_14	TTGTCACGCCGGCGGCACGGGACGCAGTAGATTCGATCAT
adpARev_14	ATGATCGAATCTACTGCGTCCCGTGCCGCCGGCGTGACAAcctaccctacgtcctcctgc
adpAFor_15	AGTAGATTCGATCATGGGTACCGAAGACTGGGGTCTCGTG
adpARev_15	CACGAGACCCCAGTCTTCGGTACCCATGATCGAATCTACTcctaccctacgtcctcctgc
adpAFor_16	GACTGGGGTCTCGTGCAAAACCGAGGGGAAACGTGCAGGA
adpARev_16	TCCTGCACGTTTCCCCTCGGTTTTGCACGAGACCCCAGTCcctaccctacgtcctcctgc
adpAFor_17	GGGAAACGTGCAGGAGCGACACGACCAGGGAGACGCGAAC
adpARev_17	GTTCGCGTCTCCCTGGTCGTGTCGCTCCTGCACGTTTCCCcctaccctacgtcctcctgc
adpAFor_18	CAGGGAGACGCGAACACCGAGGGGGGCTTAGCGTCATGAG
adpARev_18	CTCATGACGCTAAGCCCCCCTCGGTGTTCGCGTCTCCCTGcctaccctacgtcctcctgc
adpAFor_19	GCTTAGCGTCATGAGCCAGGACTCC
adpARev_19	GGAGTCCTGGCTCATGACGCTAAGCcctaccctacgtcctcctgc
MtrAFor_1	TCATATCGACATCATCCCATTTCCGTATCAGTCTCAAGGC
MtrARev_1	GCCTTGAGACTGATACGGAAATGGGATGATGTCGATATGAcctaccctacgtcctcctgc
MtrAFor_2	TATCAGTCTCAAGGCGGCTGGTGAGATACCTCACTGACCT
MtrARev_2	AGGTCAGTGAGGTATCTCACCAGCCGCCTTGAGACTGATAcctaccctacgtcctcctgc
MtrAFor_3	ATACCTCACTGACCTGCGGTGACGTCGGTGATCGCCCACC
MtrARev_3	GGTGGGCGATCACCGACGTCACCGCAGGTCAGTGAGGTATcctaccctacgtcctcctgc
MtrAFor_4	CGGTGATCGCCCACCGTCGACTGCCGGTGTCCGTGGGTGT
MtrARev_4	ACACCCACGGACACCGGCAGTCGACGGTGGGCGATCACCGcctaccctacgtcctcctgc

MtrAFor_5	GGTGTCCGTGGGTGTTGATGCCAGACATGGATGTCACCCC
MtrARev_5	GGGGTGACATCCATGTCTGGCATCAACACCCACGGACACCcctaccctacgtcctcctgc
MtrAFor_6	CATGGATGTCACCCCCGTGGGCCCCCGTGGTCGGCCTGCA
MtrARev_6	TGCAGGCCGACCACGGGGGCCCACGGGGGTGACATCCATGcctaccctacgtcctcctgc
MtrAFor_7	ACCCCCGTGGGCCCCCGTGGTCGGCCTGCACCGTACCCTG
MtrARev_7	CAGGGTACGGTGCAGGCCGACCACGGGGGCCCACGGGGGTcctaccctacgtcctcctgc
dnaAFor_1	TAGGTGGACGACGAGGAGCGGGCGACCGGCCCGGCGCCCT
dnaARev_1	AGGGCGCCGGGCCGGTCGCCCGCTCCTCGTCGTCCACCTAcctaccctacgtcctcctgc
dnaAFor_2	CCGGCCCGGCGCCCTCGGCGTACCGCGGTTGCGAAGTCCT
dnaARev_2	AGGACTTCGCAACCGCGGTACGCCGAGGGCGCCGGGCCGGcctaccctacgtcctcctgc
dnaAFor_3	CGGTTGCGAAGTCCTCGCGCCGCCTCAGCCGATTGTCGGT
dnaARev_3	ACCGACAATCGGCTGAGGCGGCGCGAGGACTTCGCAACCGcctaccctacgtcctcctgc
dnaAFor_4	CAGCCGATTGTCGGTAGGCAGCACGTCATGACCCGTTTAG
dnaARev_4	CTAAACGGGTCATGACGTGCTGCCTACCGACAATCGGCTGcctaccctacgtcctcctgc
dnaAFor_5	TCATGACCCGTTTAGCGGATCAGGCGGACAGCTCAGAGCG
dnaARev_5	CGCTCTGAGCTGTCCGCCTGATCCGCTAAACGGGTCATGAcctaccctacgtcctcctgc
dnaAFor_6	GGACAGCTCAGAGCGACCCTTGGAACGACGGTTCGCAAGG
dnaARev_6	CCTTGCGAACCGTCGTTCCAAGGGTCGCTCTGAGCTGTCCcctaccctacgtcctcctgc
dnaAFor_7	CGACGGTTCGCAAGGATGGCTCGGCCGGCACGCGTACGCA
dnaARev_7	TGCGTACGCGTGCCGGCCGAGCCATCCTTGCGAACCGTCGcctaccctacgtcctcctgc
dnaAFor_8	CGGCACGCGTACGCATCCGCAGGCGGAAGCCGTGGGTCTT
dnaARev_8	AAGACCCACGGCTTCCGCCTGCGGATGCGTACGCGTGCCGcctaccctacgtcctcctgc
dnaAFor_9	GAAGCCGTGGGTCTTGGCGCGACGACGGTTGTTCGGCTGG
dnaARev_9	CCAGCCGAACAACCGTCGTCGCGCCAAGACCCACGGCTTCcctaccctacgtcctcctgc
dnaAFor_10	CGGTTGTTCGGCTGGAAGGTGCGCTTGCTCACTCGGGGGC
dnaARev_10	GCCCCCGAGTGAGCAAGCGCACCTTCCAGCCGAACAACCGcctaccctacgtcctcctgc
dnaAFor_11	TGCTCACTCGGGGGCTCCAGAAATGATTCGTAGATGGCGG
dnaARev_11	CCGCCATCTACGAATCATTTCTGGAGCCCCCGAGTGAGCAcctaccctacgtcctcctgc
dnaAFor_12	ATTCGTAGATGGCGGGACATCGCCTGGCTGTCACCGTGCG
dnaARev_12	CGCACGGTGACAGCCAGGCGATGTCCCGCCATCTACGAATcctaccctacgtcctcctgc
dnaAFor_13	GGCTGTCACCGTGCGCCCACGAGTAGCTCGCAATACGCCC
dnaARev_13	GGGCGTATTGCGAGCTACTCGTGGGCGCACGGTGACAGCCcctaccctacgtcctcctgc
dnaAFor_14	GCTCGCAATACGCCCGAGTGCACCGCTTCACGATCACTGA

dnaARev_14	TCAGTGATCGTGAAGCGGTGCACTCGGGCGTATTGCGAGCcctaccctacgtcctcctgc
dnaAFor_15	CTTCACGATCACTGACCGTGATCTTTGCCCATCGGAGGCA
dnaARev_15	TGCCTCCGATGGGCAAAGATCACGGTCAGTGATCGTGAAGcctaccctacgtcctcctgc
dnaAFor_16	TGCCCATCGGAGGCAGGCGGCAGCAGCCATCGACAACTCG
dnaARev_16	CGAGTTGTCGATGGCTGCTGCCGCCTGCCTCCGATGGGCAcctaccctacgtcctcctgc
dnaAFor_17	GCCATCGACAACTCGACCTGGTTACGGTACGCGCGGCTAC
dnaARev_17	GTAGCCGCGCGTACCGTAACCAGGTCGAGTTGTCGATGGCcctaccctacgtcctcctgc
dnaAFor_18	GGTACGCGCGGCTACGCCATCCGGTCAAACCGACCTGTCG
dnaARev_18	CGACAGGTCGGTTTGACCGGATGGCGTAGCCGCGCGTACCcctaccctacgtcctcctgc
dnaAFor_19	CAAACCGACCTGTCGCCACCCCCCATTGTGCACAGGCTGT
dnaARev_19	ACAGCCTGTGCACAATGGGGGGTGGCGACAGGTCGGTTTGcctaccctacgtcctcctgc
dnaAFor_20	TTGTGCACAGGCTGTGGACAACAACTTGAACCACGTCATC
dnaARev_20	GATGACGTGGTTCAAGTTGTTGTCCACAGCCTGTGCACAAcctaccctacgtcctcctgc
dnaAFor_21	TTGAACCACGTCATCCGGCGCGACTACCGTGGATGGACTC
dnaARev_21	GAGTCCATCCACGGTAGTCGCGCCGGATGACGTGGTTCAAcctaccctacgtcctcctgc
dnaAFor_22	ACCGTGGATGGACTCCACAATCTTTTCCGTTCTGTCCTTA
dnaARev_22	TAAGGACAGAACGGAAAAGATTGTGGAGTCCATCCACGGTcctaccctacgtcctcctgc
dnaAFor_23	TCCGTTCTGTCCTTACCTGTCCTCACGGGTTCTGTCCTCA
dnaARev_23	TGAGGACAGAACCCGTGAGGACAGGTAAGGACAGAACGGAcctaccctacgtcctcctgc
dnaAFor_24	CGGGTTCTGTCCTCACGGACATCGACCCACCGTCCCCGAG
dnaARev_24	CTCGGGGACGGTGGGTCGATGTCCGTGAGGACAGAACCCGcctaccctacgtcctcctgc
dnaAFor_25	CCCACCGTCCCCGAGAACCACACCATCAGGGGACCTGCGA
dnaARev_25	TCGCAGGTCCCCTGATGGTGTGGTTCTCGGGGACGGTGGGcctaccctacgtcctcctgc
dnaAFor_26	TCAGGGGACCTGCGAGAAAGCGTGCCCTGTGGCTGACGTT
dnaARev_26	AACGTCAGCCACAGGGCACGCTTTCTCGCAGGTCCCCTGAcctaccctacgtcctcctgc
dnaAFor_27	CCTGTGGCTGACGTTCCTGCTGATCTTGCCGCAG
dnaARev_27	CTGCGGCAAGATCAGCAGGAACGTCAGCCACAGGcctaccctacgtcctcctgc
bldMFor_1	TTGCGCTTGTCCCCTTGAAACCGTGTCGAACGGATAGGTG
bldMRev_1	CACCTATCCGTTCGACACGGTTTCAAGGGGACAAGCGCAAcctaccctacgtcctcctgc
bldMFor_2	TCGAACGGATAGGTGGGCGGGTGGATGGCTGATGGCCGGG
bldMRev_2	CCCGGCCATCAGCCATCCACCCGCCCACCTATCCGTTCGAcctaccctacgtcctcctgc
bldMFor_3	TGGCTGATGGCCGGGCTCCTCGGGGCCCCGCCGGTCCGGG

bldMRev_3	CCCGGACCGGCGGGGCCCCGAGGAGCCCGGCCATCAGCCAcct accctacgtcctcctgc
bldMFor_4	CCCCGCCGGTCCGGGTCGGTACACCTACTGTCTAAGTAGA
bldMRev_4	TCTACTTAGACAGTAGGTGTACCGACCCGGACCGGCGGGGccta ccctacgtcctcctgc
bldMFor_5	TACTGTCTAAGTAGATGTAAATATGACTCATTGCGAATCT
bldMRev_5	AGATTCGCAATGAGTCATATTTACATCTACTTAGACAGTAcctac cctacgtcctcctgc
bldMFor_6	ACTCATTGCGAATCTAGCCACAGACACCGCGAAAAGGGAA
bldMRev_6	TTCCCTTTTCGCGGTGTCTGTGGCTAGATTCGCAATGAGTcctacc ctacgtcctcctgc
bldMFor_7	ACCGCGAAAAGGGAAGAAAACCCGCTAAATGGGGCATAGC
bldMRev_7	GCTATGCCCCATTTAGCGGGTTTTCTTCCCTTTTCGCGGTcctacc ctacgtcctcctgc
bldMFor_8	TAAATGGGGCATAGCTTTCGATGAACGACAGAAGGCTCGC
bldMRev_8	GCGAGCCTTCTGTCGTTCATCGAAAGCTATGCCCCATTTAcctac cctacgtcctcctgc
bldMFor_9	CGACAGAAGGCTCGCGTCGCTCTCCTCTGTACGCGCCCCC
bldMRev_9	GGGGGCGCGTACAGAGGAGAGCGACGCGAGCCTTCTGTCGcct accctacgtcctcctgc
bldMFor_10	TCTGTACGCGCCCCCTCACGTAGAGTGCCGAAGGGTGCCG
bldMRev_10	CGGCACCCTTCGGCACTCTACGTGAGGGGGCGCGTACAGAccta ccctacgtcctcctgc
bldMFor_11	TGCCGAAGGGTGCCGTCCGACCCGTAACTCTTTCGAGTGA
bldMRev_11	TCACTCGAAAGAGTTACGGGTCGGACGGCACCCTTCGGCAccta ccctacgtcctcctgc
bldMFor_12	AACTCTTTCGAGTGACCGTCGTTGAGAGTGCGGAGGCGGT
bldMRev_12	ACCGCCTCCGCACTCTCAACGACGGTCACTCGAAAGAGTTccta ccctacgtcctcctgc
bldMFor_13	GAGTGCGGAGGCGGTTGAAGGAACAAGCGATCGGGCAGGT
bldMRev_13	ACCTGCCCGATCGCTTGTTCCTTCAACCGCCTCCGCACTCcctacc ctacgtcctcctgc
bldMFor_14	AGCGATCGGGCAGGTGTCCGAGAGCGTCAATCGCACAGGT
bldMRev_14	ACCTGTGCGATTGACGCTCTCGGACACCTGCCCGATCGCTcctac cctacgtcctcctgc
bldMFor_15	GTCAATCGCACAGGTGACGATTACGTACAGCCCTGGAGGC
bldMRev_15	GCCTCCAGGGCTGTACGTAATCGTCACCTGTGCGATTGACcctac cctacgtcctcctgc
bldMFor_16	TACAGCCCTGGAGGCTCAAGGTGACGCGCTACAGCTGCGA
bldMRev_16	TCGCAGCTGTAGCGCGTCACCTTGAGCCTCCAGGGCTGTAcctac cctacgtcctcctgc
bldMFor_17	GCGCTACAGCTGCGAGAGCCGCGGAGGTCAGGCATGACAT
bldMRev_17	ATGTCATGCCTGACCTCCGCGGCTCTCGCAGCTGTAGCGCcctac cctacgtcctcctgc
bldMFor_18	GGTCAGGCATGACATCCGTTCTCG
bldMRev_18	CGAGAACGGATGTCATGCCTGACCcctaccctacgtcctcctgc
filPFor_1	GGGTAGCCACTCAAAAGGCTGGACATTCTCCAGATCAAAC
filPRev_1	GTTTGATCTGGAGAATGTCCAGCCTTTTGAGTGGCTACCCcctac cctacgtcctcctgc

filPFor_2	TTCTCCAGATCAAACGGGCATACGCTCGATGACACGCCGC
filPRev_2	GCGGCGTGTCATCGAGCGTATGCCCGTTTGATCTGGAGAAccta ccctacgtcctcctgc
filPFor_3	TCGATGACACGCCGCTTCGGCCCCTAGGATTCCCTCTAAC
filPRev_3	GTTAGAGGGAATCCTAGGGGCCGAAGCGGCGTGTCATCGAcct accctacgtcctcctgc
filPFor_4	AGGATTCCCTCTAACACCTCACCGGTCTCATTCGACAGGA
filPRev_4	TCCTGTCGAATGAGACCGGTGAGGTGTTAGAGGGAATCCTccta ccctacgtcctcctgc
filPFor_5	TCTCATTCGACAGGAAACCCATGAG
filPRev_5	CTCATGGGTTTCCTGTCGAATGAGAcctaccctacgtcctcctgc
whiIFor_1	AGGGGCTGTTTTCGGCCTGTTCCGGTGTCCCTCGTGGGGG
whiIRev_1	CCCCCACGAGGGACACCGGAACAGGCCGAAAACAGCCCCTcct accctacgtcctcctgc
whiIFor_2	TGTCCCTCGTGGGGGTGACGGAGCGGGGGCCGCTCGTAGG
whiIRev_2	CCTACGAGCGGCCCCCGCTCCGTCACCCCCACGAGGGACAccta ccctacgtcctcctgc
whiIFor_3	GGGGCCGCTCGTAGGATGATCGATCACGCGTCCGAATTGC
whiIRev_3	GCAATTCGGACGCGTGATCGATCATCCTACGAGCGGCCCCccta ccctacgtcctcctgc
whiIFor_4	ACGCGTCCGAATTGCCCTAATTGTTACTCACCAAATCGTG
whiIRev_4	CACGATTTGGTGAGTAACAATTAGGGCAATTCGGACGCGTccta ccctacgtcctcctgc
whiIFor_5	ACTCACCAAATCGTGATCTTTCCCTAAAGGCGGGCGGCCC
whiIRev_5	GGGCCGCCCGCCTTTAGGGAAAGATCACGATTTGGTGAGTccta ccctacgtcctcctgc
whiIFor_6	AAAGGCGGGCGGCCCAGCTGCCGAAGGAGTCAGTGACCCC
whiIRev_6	GGGGTCACTGACTCCTTCGGCAGCTGGGCCGCCCGCCTTTcctac cctacgtcctcctgc
whiIFor_7	GGAGTCAGTGACCCCTTCACAGCACGGGTTCGCCCCCGGC
whiIRev_7	GCCGGGGGCGAACCCGTGCTGTGAAGGGGTCACTGACTCCccta ccctacgtcctcctgc
whiIFor_8	GGGTTCGCCCCCGGCTTCTTCCCCGAGCCGGCTCCGTCCC
whiIRev_8	GGGACGGAGCCGGCTCGGGGAAGAAGCCGGGGGCGAACCCcc taccctacgtcctcctgc
whiIFor_9	AGCCGGCTCCGTCCCGCACCCTTCCCCCCAGGAGGCCTGG
whiIRev_9	CCAGGCCTCCTGGGGGGAAGGGTGCGGGACGGAGCCGGCTcct accctacgtcctcctgc
whiIFor_10	CCCCAGGAGGCCTGGTGTCCGTTCT
whiIRev_10	AGAACGGACACCAGGCCTCCTGGGGcctaccctacgtcctcctgc
Binding_site_T 1A	CCCTCACCCCCCACCCCTCACTCGTGTTCCCCGCGCGCTC
Binding_site_T 1A	GAGCGCGCGGGGAACACGAGTGAGGGGTGGGGGGTGAGGG cctaccctacgtcctcctgc
Binding_site_C 4G	CCCTCACCCCCCACCCCTCACTCCTGATCCCCGCGCGCTC
Binding_site_C 4G	GAGCGCGCGGGGATCAGGAGTGAGGGGTGGGGGGTGAGGG cctaccctacgtcctcctgc

Binding_site_BOTH	CCCTCACCCCCCACCCCTCACTCCTGTTCCCCGCGCGCTC
Binding_site_BOTH	GAGCGCGCGGGGAACAGGAGTGAGGGGTGGGGGGTGAGGGcc taccctacgtcctcctgc
